# PTEN-mTORC2 signaling module controls antibody isotype selection and antiviral humoral immunity

**DOI:** 10.3389/fimmu.2026.1771230

**Published:** 2026-02-10

**Authors:** Bikash Thapa, Yejin Lee, Seongwon Pak, Dongkyu Kim, Hyung-Joo Kwon, Keunwook Lee

**Affiliations:** 1 Institute of Bioscience & Biotechnology, Hallym University, Chuncheon, Republic of Korea; 2 Department of Biomedical Science, Hallym University, Chuncheon, Republic of Korea; 3 Department of Microbiology, College of Medicine, Hallym University, Chuncheon, Republic of Korea

**Keywords:** antibody isotype selection, influenza A virus, mTORC2, Notch, phosphoinositide 3-kinase pathway, PTEN

## Abstract

**Introduction:**

The phosphoinositide 3-kinase (PI3K) pathway is a central regulator of activated B cells, yet how PI3K signal strength is interpreted within complex signaling networks to achieve appropriate antibody isotype selection and protective humoral immunity remains incompletely understood. Building on prior work implicating mTOR complexes in germinal center (GC) reactions, we investigated how this circuitry operates under pathological PI3K hyperactivation driven by loss of the PI3K antagonist PTEN.

**Methods:**

Using *in vitro* GC-like B cell cultures and conditional knockout mouse models, we examined the roles of mTORC1 and mTORC2 downstream of PTEN loss. Transcriptomic profiling, pharmacologic perturbation of Akt and Notch signaling, and *in vivo* immunization and passive influenza A virus protection assays were employed to define functional and mechanistic outcomes.

**Results:**

PTEN deficiency impaired IgG1 class switching, a defect selectively rescued by genetic inactivation of mTORC2 but not mTORC1. mTORC2 ablation in PTEN-deficient B cells restored *Aicda* expression and promoted IgG1⁺ plasmablast differentiation without enhancing IgE switching. Transcriptomic analyses revealed that the PTEN–mTORC2 signaling module supports transcriptional programs associated with GC organization, isotype switching, and secretory capacity. Functionally, disruption of mTORC2 in PTEN-deficient B cells reinstated isotype-switched antibody responses, including IgG1, IgG3, and IgA, and restored antibody-mediated protection against influenza A virus infection in a passive immunization setting. Mechanistically, pharmacologic and expression-based perturbations suggested that mTORC2–Akt signaling can cooperate with the Notch pathway to fine-tune IgG1 versus IgE isotype selection.

**Conclusion:**

Together, these findings define a PTEN–mTORC2–Akt signaling module within the PI3K network that shapes antibody isotype selection and antiviral humoral immunity, highlighting a potential target for precision modulation of antibody responses in infection, vaccination, and inflammatory diseases.

## Introduction

The humoral immune system protects the host from invading pathogens through antibody-mediated mechanisms, including neutralization, phagocytosis, and complement activation. Robust and durable antibody responses rely on the formation of germinal centers (GCs), where activated B cells undergo somatic hypermutation (SHM) and class switch recombination (CSR) to generate high-affinity, isotype-switched antibodies (1). SHM introduces point mutations into the immunoglobulin (Ig) variable regions to enhance antigen-binding affinity, whereas CSR replaces the IgM constant region with alternative isotypes (*e.g.*, IgG1, IgE, IgA), thereby tailoring antibody effector function (2, 3). Both processes are initiated by activation-induced cytidine deaminase (AID) (4, 5). For example, AID targets the switch regions of Ig heavy chain loci to facilitate CSR via non-homologous end-joining. Loss-of-function mutations in *AICDA* result in hyper-IgM syndrome, whereas aberrant AID expression contributes to genomic instability and lymphomagenesis (6–8).

Activated B cells integrate cues from the B cell receptor (BCR), CD40, and cytokine receptors via intracellular signaling pathways to coordinate clonal expansion, selection, and terminal differentiation (1). Among these, the phosphoinositide 3-kinase (PI3K) pathway is essential for GC B cell survival, metabolic fitness, and fate specification (9). However, accumulating evidence indicates that excessive or sustained PI3K signaling paradoxically disrupts GC organization and compromises antibody diversification (10, 11). The lipid phosphatase PTEN antagonizes PI3K signaling, and PTEN loss in B cells leads to reduced AID expression, defective CSR and SHM, culminating in a hyper-IgM-like phenotype (12, 13). These observations underscore that signal intensity and context are critical determinants of downstream functional outcomes in antibody responses of activated B cells.

Downstream of PI3K, Akt and mammalian target of rapamycin (mTOR) function as central signaling hubs that integrate mitogenic, metabolic, and survival cues in B cells. mTOR exists in two functionally distinct complexes, mTORC1 and mTORC2. mTORC1 promotes anabolic metabolism and clonal expansion of activated B cells, whereas sustained mTORC1 activity disrupts GC dark zone–light zone (DZ–LZ) organization and compromises GC selection and affinity maturation (14–16). mTORC2 phosphorylates Akt at Ser473, thereby enhancing kinase activity and altering substrate specificity, and has been shown to be required for mature B cell homeostasis and antibody responses (17). Notably, elevated Akt signaling has been associated with reduced AID expression and impaired CSR efficiency (12, 13). Moreover, FoxO1, a transcriptional factor negatively regulated by Akt, is essential for DZ maintenance and *Aicda* transcription during GC reactions (11, 18). Thus, loss of PTEN enforces sustained PI3K–Akt activity, potentially limiting FoxO1 function and suppressing CSR. Collectively, these studies established that PI3K signaling strength must be precisely controlled to support effectively antibody diversification.

Importantly, several reports have demonstrated that perturbations of PTEN, Akt or mTOR signaling impair CSR and SHM, and that mTORC1 and mTORC2 exert nonredundant and sometimes opposing roles in GC B cell biology and antibody responses (13, 15–17, 19). However, despite this substantial body of work, how individual PI3K pathway components are coordinately engaged to calibrate isotype selection and GC output within the context of PI3K signaling network remains incompletely understood. This gap persists in part because global disruption of mTOR signaling often compromises B cell survival and proliferation, complicating functional interpretation of downstream effects on antibody quality (20–22). Although previous studies revealed a dynamic modulation of PI3K signaling strength across GC zones and stages of the response (16, 23), how this network is optimally tuned to generate high-affinity, isotype-switched antibodies remains unresolved.

Recent translational observations further underscore the clinical relevance of PI3K-mTOR pathway regulation in humoral immunity. For example, mTOR inhibitors administered to kidney transplant recipients have been associated with enhanced neutralizing IgG responses following SARS-CoV-2 mRNA vaccination (24, 25). Conversely, patients with autoimmune lymphoproliferative syndrome (ALPS) harboring *FAS* mutations display elevated PI3K-mTOR signaling and defective GC formation, potentially linked to altered PTEN function (26). Notably, prior work has demonstrated that mTORC2 restrains IgG1 switching under physiological activation conditions, whereas mTORC1 supports key aspects of the CSR program (15, 19). These findings provide an important framework but leave open how this circuitry operates when PI3K signaling is amplified. In this study, we build on this foundation by examining how mTORC1 and mTORC2 function within a PTEN-deficient, hyper-PI3K signaling context, which represents a condition relevant to immunodeficiency, autoimmunity, and dysregulated vaccine responses. Using *in vitro* GC-like B cell cultures and conditional genetic models targeting PTEN, Raptor (mTORC1), and Rictor (mTORC2), we define a PTEN-mTORC2-Akt signaling module that selectively constrains IgG1 class switching under PI3K hyperactivation. We further show that targeted modulation of this signaling module restores isotype-switched antibody production and antibody-mediated protection against influenza A virus. Finally, our data suggest that Notch signaling serves as an auxiliary modulatory input within the PI3K network, contributing to the fine-tuning of antibody isotype selection. Together, our findings position PTEN-mTORC2-Akt signaling as a critical node within the broader PI3K network that calibrates antibody isotype selection and humoral immune quality.

## Materials and methods

### Mice

*Pten*^fl/fl^, *Pten*^fl/fl^:*Raptor*^fl/fl^, and *Pten*^fl/fl^:*Rictor*^fl/fl^ mice were crossed with *ROSA26*^Cre-ERT2^ mice. To induce deletion of conditional alleles, mice were treated intraperitoneally with tamoxifen (3 mg per dose) on two consecutive days, as previously described (27). Splenic B cells were isolated five days after the third injection for *in vitro* experiments or adoptive transfer, allowing sufficient time for Cre-mediated recombination and depletion of pre-existing PTEN Raptor, or Rictor protein. Mice are hereafter referred to as *Pten*^δ^, *Pten*^δ^:*Raptor*^δ^, and *Pten*^δ^:*Rictor*^δ^, respectively. For deletion in the AID-expressing B cell compartment, these strains were crossed with *Aicda*^Cre^ mice to generate *Aicda*^Cre/+^:*Pten*^fl/fl^ and *Aicda*^Cre/+^:*Pten*^fl/fl^:*Rictor*^fl/fl^, hereafter referred to as *Pten*^B^ and *Pten*^B^*: Rictor*^B^, respectively. Control *ROSA26*^Cre-ERT2^ and *Aicda*^Cre/+^ littermates or age-matched controls were used throughout. *Pten*^fl/fl^, *Raptor*^fl/fl^, *Rictor*^fl/fl^ and *ROSA26*^Cre-ERT2^ mice were provided by M. Boothby (Vanderbilt University). *Aicda*^Cre/Cre^ and *Rag2*^-/-^ mice were purchased from Jackson Laboratory. C57BL/6J mice were obtained from DBL Korea. All mice were housed under specific pathogen-free conditions at the Laboratory Animal Center of Hallym University. Influenza A virus (IAV) infection experiments were conducted under biosafety level 2 (BSL-2) at the Medical and Bio-Convergence Research Institute of Hallym University. All animal procedures complied with guidelines from the Korean Ministry of Food and Drug Safety and were approved by the Institutional Animal Care and Use Committee of Hallym University [Hallym 2018-58; Hallym 2020-47; Hallym 2023-55].

### Purification of B cells, GC B cells and CD4 T cells

Single-cell suspensions from spleens or lymph nodes were prepared as described (28). B cells were isolated by depleting CD43^+^ cells using CD43 (Ly-48) MicroBeads and MACS Separator (Miltenyi Biotec). GC B cells were purified using the Mouse Germinal Center B cell MicroBead Kit (Miltenyi Biotec) according to the manufacturer’s instructions. CD4 T cells were isolated using CD4 (L3T4) MicroBeads (Miltenyi Biotec).

### iGCB and iPB culture

CD40LB feeder cells that express murine CD40L and B cell activation factor (BAFF) (kind gift from S.G. Kang, Kangwon National University) were cultured in DMEM containing 10% FBS (29). Feeder cells were plated in 60-mm dishes, treated with 7 µM mitomycin C (Sigma) for 3 h, and washed several times to remove residual mitomycin C. Naïve B cells were co-cultured with CD40LB feeder cells in complete RPMI-1640 (Welgene) supplemented with 2 ng/ml mIL-4 (R&D Systems) to generate *in vitro* GC-like B cells (iGCBs) over 4 days. iGCBs were then harvested and cultured with mitomycin C-treated CD40LB cells in the presence of 10 ng/ml mIL-21 (PeproTech) for an additional four days to induce plasmablast (iPB) differentiation. In some experiments, iGCB cultures were treated with 5 nM Rapamycin (Tocris), 25 nM Torin-2 (Tocris), 1 μM MK-2206 (Selleckchem), or 50 μM DAPT (MCE).

### Flow cytometry

Single-cell suspensions were prepared from iGCB and iPBs cultures and spleen and incubated with Fc Block (BD Bioscience). Cells were stained with fluorescence-conjugated antibodies in the presence of 7-AAD (ThermoFisher) for viability exclusion, as previously described (28). The following antibodies were used: CD19 (1D3), B220 (RA3-6B2), GL7 (GL7), Fas (Jo2), CD138 (clone 281-2), CXCR4 (clone 2B11), IgM (II/41), IgD (11-26c.2a), IgG1 (A85-1), and IgE (RME-1). Flow cytometry antibodies were purchased from BD Bioscience, ThermoFisher, or BioLegend. Data were acquired on a BD FACS Canto II instrument using FACSDiva software and analyzed with FlowJo software V10.8.1 (BD Bioscience).

### Adoptive transfer and immunization

A fraction of 12 x 10^6^ B cells isolated from tamoxifen-treated *ROSA26*^Cre-ERT2^:*Pten*^fl/fl^, *ROSA26*^Cre-ERT2^:*Pten*^fl/fl^:*Raptor*^fl/fl^, *ROSA26*^Cre-ERT2^:*Pten*^fl/fl^:*Rictor*^fl/fl^, or control mice were mixed with 4 x 10^6^ wild-type CD4^+^ T cells and intravenously transferred into *Rag2*^-/-^ mice. Mice were immunized intraperitoneally with 50 µg 4-hydroxy-3-nitrophenylacetyl (NP)-conjugated ovalbumin (OVA) emulsified in alum and boosted with NP-OVA in PBS 14 days later to ensure robust, synchronized germinal center responses in the adoptive transfer setting. Sera were collected 5 days after the boost immunization. To examine GC reactions *in vivo*, *Aicda*^Cre/+^:*Pten*^fl/fl^, *Aicda*^Cre/+^:*Pten*^fl/fl^:*Rictor*^fl/fl^, and control mice were injected intraperitoneally with 0.2 ml of a 100% sRBC suspension (Innovative research). Splenic GC B cells, plasma cells and Ig isotype switching were analyzed by flow cytometry on day 9 post-immunization.

### Influenza A virus infection

The H1N1 mouse-adapted IAV strain A/WSN/1933 was provided by Hyung-Joo Kwon (Hallym University). Virus stocks were prepared in embryonated chicken eggs under BSL-2 conditions and quantified by plaque assay (30). 8–10 week-old male *Aicda*^Cre/+^:*Pten*^fl/fl^, *Aicda*^Cre/+^:*Pten*^fl/fl^:*Rictor*^fl/fl^, and control mice were immunized intraperitoneally with 1 × 10^7^ pfu WSN virus. After 20 days, mice were intranasally infected with 1 × 10^6^ pfu WSN. For passive immunization, sera were collected 21 days post-immunization, pooled, and 100 μl sera were injected intravenously into C57BL/6J males, followed by intranasal infection WSN infection. Viral titers in lungs were assessed at 6 days post-infection by plaque assay or quantitative real-time PCR detecting the WSN nucleocapsid protein (NP) gene. Lung tissues were harvested after perfusion and fixed with 4% paraformaldehyde for histological analysis with hematoxylin and eosin staining (Merck). Images were acquired using an Eclipse Ni-U upright microscope (Nikon).

### ELISA

Relative levels of serum NP-specific antibodies were measured by capture ELISA using NP_30_-BSA-coated plates, as described (28). Anti-NP IgM, IgG1, IgG2b, IgG3 and IgA were detected using SBA Clonotyping System-HRP kit (SouthernBiotech). High-affinity and cross-reactive antibodies were assessed using NP_2_-BSA or 4-hydroxy-3-iodo-5-nitrophenylacetyl (NIP)-BSA as capture antigens, respectively. Anti-IAV antibodies were detected using plates coated with 1 x 10^6^ pfu WSN virus.

### RNA-seq analysis

Total RNA was extracted from iGCBs using TRIzol (Invitrogen). Libraries were prepared using the TruSeq Standard mRNA Library Prep Kit (Illumina) according to the manufacturer’s instructions. Sequencing was performed on an Illumina NovaSeq 6000 platform by Macrogen. Sequencing reads were aligned to the *Mus musculus* reference genome mm10 using HISAT2 v2.1.0. and assembled with StringTie v2.1.3b. Differentially expressed genes (DEGs) were identified using DESeq2 with threshold set a false discovery rate (FDR) < 0.05 and fold change > 1.5. Functional annotation of DEGs was conducted using DAVID Bioinformatics Resources (https://davidbioinformatics.nih.gov), and pathway enrichment was analyzed using the Kyoto Encyclopedia of Gens and Genomes (KEGG) database within DAVID. Heatmaps were generated using the heatmap.2 in the gplots package in RStudio v3.4.4. Gene expression correlations were assessed using FPKM values and Pearson’s correlation coefficients were calculated using GraphPad Prism v7.04 (GraphPad Software).

### Reverse transcriptional PCR and western blotting

Total RNA was extracted from iGCBs or splenic GL-7^hi^ Fas^+^ B cells using TRIzol reagent (ThermoFisher) and reversed-transcribed with the ImProm-II Reverse Transcription system (Promega) according to the manufacturers’ instructions. Quantitative real-time PCR was performed using SYBR qPCR mix (Toyobo) on a CFX96 Real-Time PCR Detection System (Bio-Rad). Primer pairs are listed in **Supplementary Table 1**. Data were normalized to *Actb* in each sample and calculated using the comparative 2^-δδCT^ method (31). For immunoblotting, iGCBs lysates were prepared in RIPA lysis buffer supplemented with protease and phosphatase inhibitor cocktails (GenDEPOT). Protein lysates were separated by SDS-PAGE and transferred to PVDF membranes. Membranes were incubated with the indicated primary antibodies, followed by HRP-conjugated secondary antibodies (Enzo Life Sciences). Monoclonal antibodies against phospho-S6^Ser240/244^ (D68F8), S6 ribosomal protein (5G10), phospho-4E-BP1^Thr37/46^ (236B4), 4E-BP1 (53H11), phospho-Akt^Ser473^ (D9E), PTEN (D4.3), Raptor (24C12), phospho-GSK-3β^S9^ (D3A4), GSK-3β (D5C5Z), AID (L7E7), Hes1 (D6P2U), Notch2 (D76A6), α-tubulin (DM1A) and β-actin (8H10D10), as well as polyclonal antibodies against Akt and Rictor, were purchased from Cell Signaling Technology. Protein signals were detected using an Enhanced Chemiluminescence Detection kit (Merk Millipore).

### Statistical analysis

All data are representative of at least two independent experiments with consistent results and are presented as means ± standard deviation (S.D). Statistical analyses were performed using Prism v7.04 (GraphPad Software). Data distributions were assessed for normality using the Shapiro–Wilk test prior to parametric testing. Comparisons between two groups were performed using an unpaired two-tailed Student’s *t*-test. For experiments involving multiple comparisons, two-way analysis of variance (ANOVA) was used, followed by Bonferroni’s multiple-comparison *post hoc*. Outliers were identified and excluded using the ROUT method (*Q* = 5%). Statistically significance was reported using predefined threshold *p* values (*p* < 0.05, *p* < 0.01, *p* < 0.001), as indicated in the figure legends. Statistical tests used and the numbers of biological replicates (*n*) are specified in the corresponding figure legends.

## Results

### mTOR signaling negatively regulates IgG1 isotype switching in *in vitro* GC-like B cells

**Figure 1:**
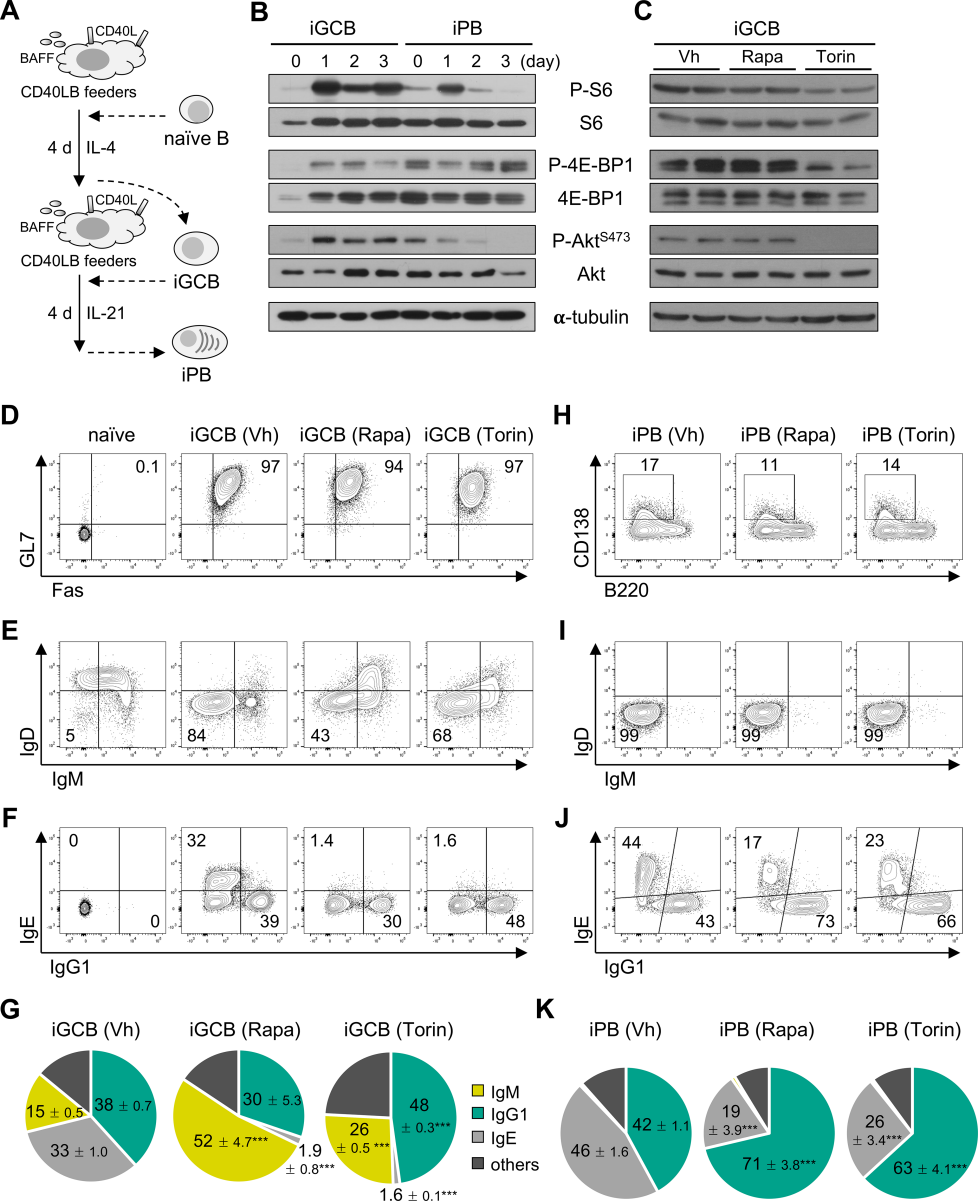
mTOR signaling negatively regulates IgG1 isotype switching. **(A)** Schematic of *in vitro* differentiation of GC-like B cells (iGCBs) and plasmablasts (iPBs). Splenic B cells were co-cultured with feeder cells expressing CD40L and BAFF (CD40LB) in the presence of IL-4 to generate iGCBs, followed by IL-21 to induce iPB differentiation. **(B)** Time-course immunoblot analysis of mTOR signaling in iGCBs and iPBs. Data shown are representative of two or three independent experiments. **(C)** Immunoblot analysis of iGCBs treated with rapamycin (Rapa, 5 nM), torin-2 (Torin, 25 nM), or vehicle (Vh). Representative immunoblots from four independent experiments are shown. **(D–F)** Representative flow cytometry (FACS) plots of iGCBs cultured in the presence of Rapa, Torin, or Vh, gated on viable B cells, with percentages of the indicated populations. **(G)** Quantification of Ig isotype distribution in iGCBs, presented as pie charts. **(H–J)** Representative FACS plots of iPBs differentiated from iGCBs treated with Rapa, Torin, or Vh, gated on viable B cells, with percentages of the indicated populations. **(K)** Quantification of Ig isotype distribution in iPBs. Data in **(D–F)** and **(H–J)** are representative of two independent experiments, each performed with three biological replicates. Statistical significance in **(G)** and **(K)** was assessed by an unpaired two-tailed Student’s t-test: ***p < 0.001.

GC reactions are orchestrated through dynamic interactions between B cells, follicular helper T cells and stromal cells to generate of high-affinity, isotype-switched antibodies, as well as long-lived plasma cells and memory B cells (1). Although mTOR signaling has been implicated in GC B cell biology, the precise regulation of mTOR activity required for optimal humoral immunity remains incompletely understood. To address this, we utilized an *in vitro* GC-like B cell (iGCB) culture system in which naïve B cells were cocultured with CD40L- and BAFF-expressing feeder cells to mimic GC-like activation *in vivo* (29) (**Figure 1A**). During iGCB activation and differentiation, we observed robust induction of both mTORC1 and mTORC2 activities, as evidenced by phosphorylation of S6 and 4E-BP1 (mTORC1) and Akt (mTORC2), respectively (**Figure 1B**; **Supplementary Figure S1a**). Notably, Akt phosphorylation declined during *in vitro* plasmablast (iPB) differentiation, whereas 4E-BP1 phosphorylation remained sustained.

To assess the functional consequences of mTOR activity, we treated iGCBs and iPBs with low doses of the mTOR inhibitors rapamycin (Rapa) and torin-2 (Torin), which were selected to minimize cytotoxic and cytostatic effects (**Supplementary Figure S1b**) (19, 32). Torin treatment efficiently suppressed phosphorylation of S6, 4E-BP1, and Akt without impairing iGCB differentiation or expression of the activation markers such as CD40 (**Figures 1C, D**; **Supplementary Figures S1c, d**). While Rapa had only a marginal effect on S6 phosphorylation, both inhibitors abrogated IL-4-induced isotype switching to IgE and increased the frequency of unswitched IgM^+^ iGCBs (**Supplementary Figure S1c**; **Figures 1E–G**). Intriguingly, Torin significantly enhanced the frequency of IgG1^+^ iGCBs, whereas Rapa had no detectable effect on IgG1 class switching (**Figures 1F, G**). Analysis of absolute cell numbers revealed that Torin treatment had minimal effects on iGCB expansion, whereas rapamycin modestly but significantly reduced iGCB viability (**Supplementary Figure S1b**), indicating that the differential effects of Torin on IgG1 switching are not attributable to major differences in cell survival or proliferation. Upon differentiation into iPBs, CD138^+^ B220^lo^ cells were comparably induced and more than 99% of iPBs had lost surface IgM and IgD expression (**Figures 1H, I**). Remarkably, both Rapa and Torin treatment promoted the emergence of IgG1^+^ iPBs at the expense of IgE^+^ cells (**Figures 1J, K**). Together, these results demonstrate that mTOR signaling acts as a negative regulator of IgG1 isotype switching in GC-like activated B cells.

### mTORC2, but not mTORC1, restricts IgG1 isotype switching in PTEN-deficient iGCBs

**Figure 2:**
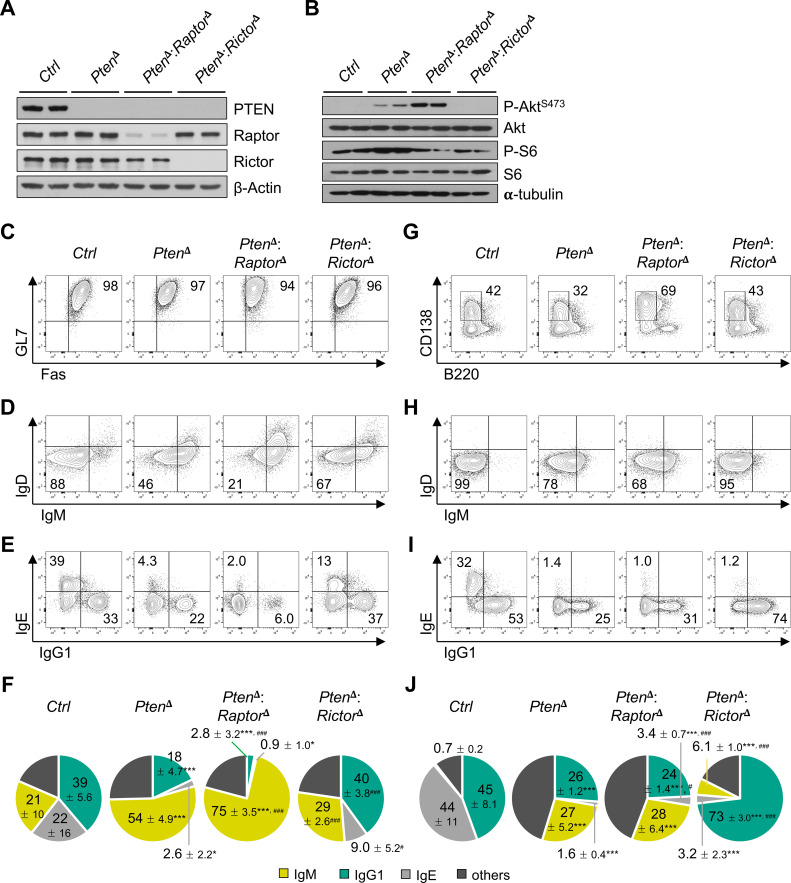
Inactivation of mTORC2 enhances IgG1 isotype switching in PTEN-deficient B cells. **(A)***ROSA26*^Cre-ERT2^:*Pten*^fl/fl^ (*Pten*^δ^), *ROSA26*^Cre-ERT2^:*Pten*^fl/fl^:*Raptor*^fl/fl^ (*Pten*^δ^:*Raptor*^δ^), *ROSA26*^Cre-ERT2^:*Pten*^fl/fl^:*Rictor*^fl/fl^ (*Pten*^δ^:*Rictor*^δ^), or control mice (*Ctrl*) were treated with tamoxifen, as describe in “Materials and Methods”, and depletion of PTEN, Raptor, and Rictor in splenic B cells was confirmed by Western blotting. **(B)** iGCBs were cultured for two days, and mTORC1 and mTORC2 activities were assessed by immunoblotting. Data shown are representative of two or three independent experiments. **(C–E)** Representative FACS profiles of iGCBs cultured as in **Figure 1**, gated on viable B cells, with percentages of the indicated population. **(F)** Relative proportions of Ig isotypes in iGCBs, presented as pie charts. **(G–I)** Representative FACS plots of iPBs differentiated from iGCBs, gated on viable B cells, with percentages of the indicated population. **(J)** Relative proportions of Ig isotypes in iPBs. Data in **(C–J)** are representative of two independent experiments, each consisting of six biological replicates: *p < 0.05 and ***p < 0.001 compared to *Ctrl*; ^#^p < 0.05 and ^###^p < 0.001 compared to *Pten*^δ^.

PI3K signaling is essential for B cell activation and differentiation, but its amplitude must be tightly regulated to support efficient CSR and affinity maturation. Loss of PTEN, a negative regulator of PI3K signaling, results in pathologic pathway hyperactivation and defective GC output (10, 13). To dissect the individual contributions of mTOR complexes within this hyperactive signaling context, we generated inducible KO mice lacking PTEN alone or in combination with either Raptor (an essential component of mTORC1) or Rictor (a regulatory subunit of mTORC2) (**Figure 2A**). Consistent with previous studies (17, 27), deletion of either Raptor or Rictor alone resulted in markedly reduced B cell viability, whereas combined deletion with PTEN did not impair expansion of iGCBs (**Supplementary Figure S2a**). This genetic framework therefore enabled direct comparison of PTEN-deficient B cells (*Pten*^δ^) with double-deficient cells (*Pten*^δ^:*Raptor*^δ^ and *Pten*^δ^:*Rictor*^δ^) to functionally interrogate the specific roles of mTORC1 and mTORC2 downstream of PI3K, while minimizing confounding effects on B cells survival and proliferation (17, 27). Western blotting analysis confirmed that PTEN loss resulted in elevated phosphorylation of both S6 and Akt, indicative of increased mTORC1 and mTORC2 activities, respectively (**Figure 2B**; **Supplementary Figure S2b**). Although Raptor depletion reduced S6 phosphorylation, residual S6 phosphorylation remained detectable, consistent with incomplete depletion of mTORC1 activity in this inducible system (**Figures 2A, B**, **Supplementary Figure S2b**). Nonetheless, phosphatidylinositol (3,4,5)-triphosphate (PIP3) levels were comparably increased across all PTEN-deficient genotypes, irrespective of Raptor or Rictor deletion (**Supplementary Figures S2c, d**), confirming persistent upstream PI3K pathway activation. Despite comparable induction of GL7^hi^ Fas^+^ iGCBs across all genotypes, the surface expression of the costimulatory molecules CD80, CD86, and CD40 was selectively reduced in *Pten*^δ^:*Raptor*^δ^ iGCBs (**Figure 2C**; **Supplementary Figure S2e**). This phenotype may reflect the functional impact of reduced, though not completely abolished, mTORC1 signaling in this context.

Functionally, PTEN deficiency impaired IL-4-induced isotype switching to both IgG1 and IgE, leading to accumulation of unswitched IgM^+^ iGCBs (**Figure 2D**). Notably, genetic deletion of Rictor selectively restored IgG1 class switching in PTEN-deficient iGCBs, whereas Raptor deletion further exacerbated the CSR defect (**Figures 2D–F**). Upon differentiation into iPBs, PTEN deficiency attenuated the generation of B220^lo^ CD138^+^ iPBs, while combined deletion of PTEN and Raptor enhanced iPB formation (**Figure 2G**). Consistent with the iGCBs phenotype, both *Pten*^δ^ and *Pten*^δ^:*Raptor*^δ^ iPBs displayed defective isotype switching, characterized by retention of IgM^+^ cells and reduced IgG1 and IgE expression (**Figures 2H–J**). In contrast, *Pten*^δ^:*Rictor*^δ^ iPBs exhibited markedly increased IgG1 switching relative to control cells (**Figures 2H–J**). Enhanced IgG1 secretion and productive recombination at the IgG1 heavy chain gene locus were further confirmed in these cells (**Supplementary Figures S2f, g**). Nonetheless, mTORC2 inactivation did not rescue IgE class switching in PTEN-deficient iGCBs or iPBs (**Figures 2F–J**). Together with our pharmacologic data, these results demonstrate that mTORC2, but not mTORC1, negatively regulate IgG1 isotype switching in the setting of hyperactivated PI3K signaling. Extending these findings beyond the GC-like culture system, we further observed that mTORC2 inactivation restored IL-4-induced IgG1 switching in PTEN-deficient B cells stimulated with LPS in a T-independent plasmablast culture (**Supplementary Figure S2h**). Collectively, these data underscore the central role of the PTEN-mTORC2 signaling axis in calibrating optimal IgG1 switching across both T-dependent and T-independent activated B cell activation contexts.

### The PTEN-mTORC2 axis controls class switching and affinity maturation of antibodies

**Figure 3:**
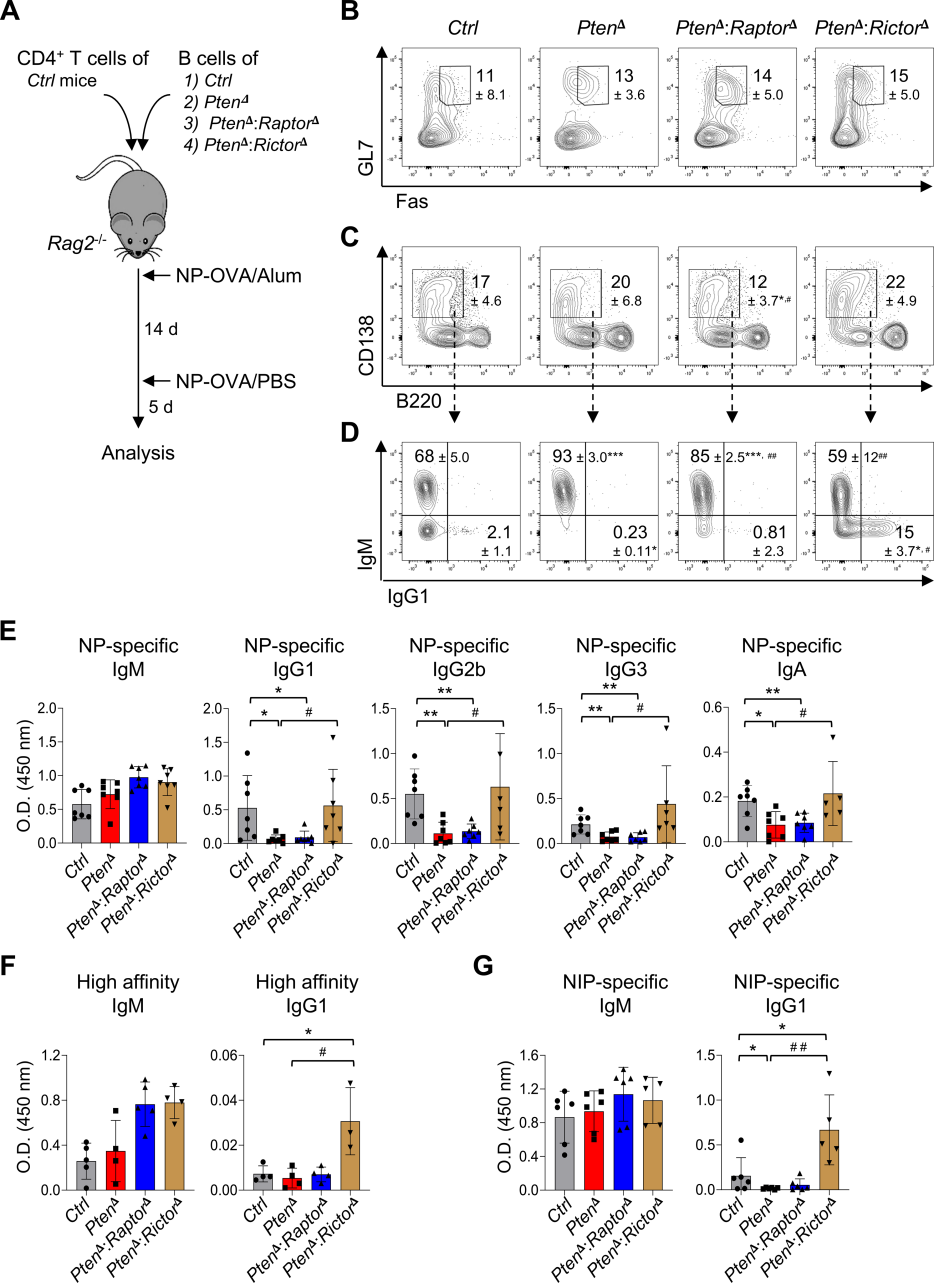
PTEN-mTORC2 axis regulates class switching and affinity maturation of antibodies. **(A)** Schematic of the adoptive transfer and immunization model. *Rag2*^-/-^ mice were reconstituted with B cells of the indicated genotypes together with wild-type CD4^+^ T cells, followed by immunization with NP-conjugated ovalbumin (NP-OVA). **(B–D)** Five days after booster immunization, splenic GL-7^hi^ Fas^+^ B cells, CD138^+^ B220^lo^ plasma cells, and Ig isotype switching were analyzed by flow cytometry. Representative FACS profiles gated on viable B cells are shown, with percentages (± SD) of the indicated populations. Data shown are representative of two independent experiments, each performed with six or eight mice per group. **(E)** Relative levels of NP-specific serum antibody isotypes were quantified by ELISA using NP_30_-BSA as the capture antigen: *n* = 7 per group. **(F, G)** High affinity NP-specific antibodies and cross-reactive antibodies binding to the structurally related hapten NIP were detected using NP_2_-BSA and NIP-BSA as capture antigens, respectively. Statistical significance was determined using two-way ANOVA with Bonferroni’s multiple-comparison correction: *p < 0.05, **p < 0.01 and ***p < 0.01 compared to *Ctrl*; ^#^p < 0.05 and ^##^p < 0.01 compared to *Pten*^δ^.

Our *in vitro* data indicated that the PTEN-mTORC2 signaling module plays a critical role in promoting IgG1 CSR under conditions of PI3K hyperactivation. To evaluate the *in vivo* relevance of this pathway, we reconstituted immunodeficient *Rag2*^-/-^ mice with B cell isolated from tamoxifen-treated inducible KO mice, together with wild type CD4^+^ T cells (**Figure 3A**). Because adoptive transfer into *Rag2*^-/-^ hosts can result in variable or heterogeneous primary GC responses, a booster immunization was included to reinforce robust, synchronized GC-dependent antibody responses at the time of analysis (15). Following immunization with NP-conjugated ovalbumin (NP-OVA), frequencies of GL7^hi^ Fas^+^ GC B cells and B220^lo^ CD138^+^ plasma cells were comparable across groups, whereas the total numbers of plasma cells were reduced in mice receiving *Pten*^δ^:*Raptor*^δ^ B cells (**Figures 3B, C**; **Supplementary Figure S3**). Consistent with our *in vitro* iPB culture data, PTEN-deficient B cells generated significantly fewer IgG1^+^ plasma cells *in vivo*. Remarkably, this defect was selectively rescued by concurrent deletion of Rictor, but not Raptor, highlighting a specific requirement for mTORC2 downregulation in IgG1 CSR (**Figure 3D**).

In line with these results, serum levels of NP-specific class-switched antibodies, including IgG1, IgG2b, IgG3, and IgA, were significantly diminished in mice reconstituted with *Pten*^δ^ B cells and were restored by mTORC2 inactivation in the PTEN-deficient background (**Figure 3E**). Notably, NP-specific IgM levels remained unchanged across all groups, indicating that the PTEN-mTORC2 axis primarily affects isotype switching rather than global antibody production. Although the frequency of IgG1^+^ plasma cells was increased in mice receiving *Pten*^δ^:*Rictor*^δ^ B cells compared with controls, NP-specific IgG1 levels in these mice was comparable to controls (**Figures 3D, E**). Quantification of absolute plasma cell numbers revealed a reduced despite variable total plasma cell pool in this group (**Supplementary Figure S3c, d**), which likely accounts for the apparent discrepancy between cellular frequencies and serum antibody levels. These findings suggest that reduced plasma cell output may contribute to the limited accumulation of antigen-specific IgG1 in this setting, despite increased frequencies of IgG1^+^ plasma cells. Importantly, high-affinity NP-specific IgG1 responses and cross-reactive binding to the structurally related hapten NIP were significantly enhanced in mice reconstituted with *Pten*^δ^:*Rictor*^δ^ B cells (**Figures 3F, G**). Together, these data demonstrate that mTORC2 inactivation can compensate for PI3K hyperactivation in PTEN-deficient B cells, effectively rescuing both CSR and affinity maturation during the humoral immune responses.

### PTEN-mTORC2 signaling dictates protective humoral responses against influenza A virus

**Figure 4:**
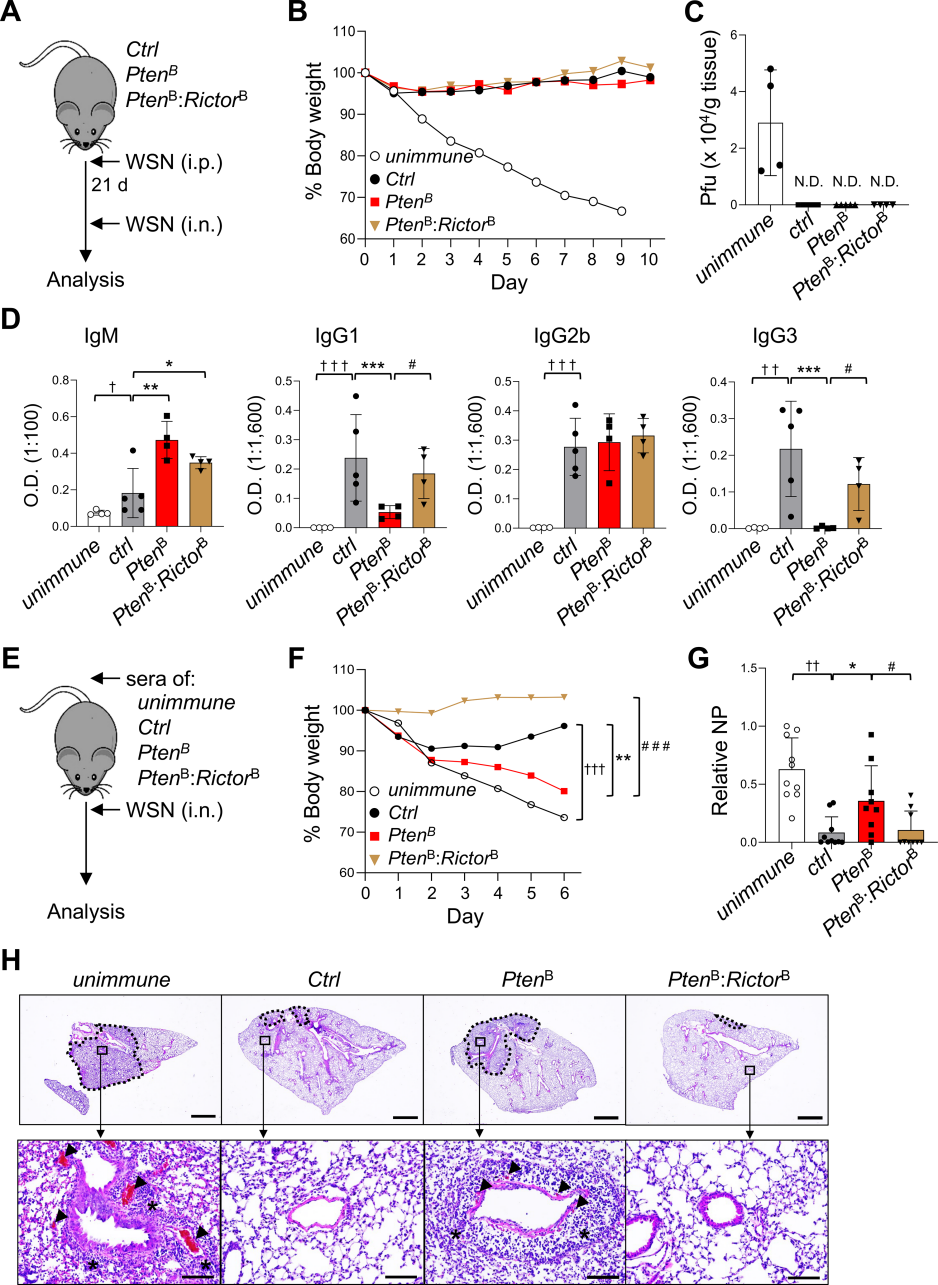
PTEN-mTORC2 modulation is required for protective humoral immunity to influenza A virus. **(A)** Schematics of the immunization and lethal influenza A/WSN virus challenge model. *Aicda*^Cre^-driven conditional KO (*Pten*^B^ and *Pten*^B^*: Rictor*^B^) or control mice (*Ctrl*) were immunized intraperitoneally with WSN virus and challenged intranasally with a lethal dose of the same virus three weeks post-immunization. **(B, C)** Body weight loss and lung viral loads were determined. Data shown are representative of two independent experiments, each performed with six mice per group. **(D)** Sera were collected three weeks post-immunization, and virus-specific antibody levels were quantified by ELISA using whole viral lysate as the capture antigen: *n* = 5 per group. **(E)** Passive immunization and viral challenge model. *Pten*^B^, *Pten*^B^*: Rictor*^B^, or control mice were immunized with WSN as in **(A)**, and the immune sera was transferred into naïve C57BL/6J recipients, followed by lethal intranasal challenge with WSN virus. **(F, G)** Body weight loss and lung viral load were measured. Data shown are representative of two independent experiments, each performed with twelve mice per group. Statistical significance was determined using two-way ANOVA with Bonferroni’s multiple-comparison correction: ^†^p < 0.05, ^††^p < 0.01 and ^†††^p < 0.001 compared to unimmunized control, *p < 0.05, **p < 0.01 and ***p < 0.001 compared to *Ctrl*; ^#^p < 0.05 and ^##^p < 0.01 compared to *Pten*^δ^. **(H)** Lung histopathology was analyzed by H&E staining: dotted line, inflamed lesion; arrowheads, hemorrhage; asterisks, immune cell infiltrates; scale bar = 2 mm (upper panels) and 100 μm (lower panels). Images are representative of six mice per group.

Given the requirement for tightly regulated PI3K signaling in orchestrating effective antibody responses, we next assessed the functional relevance of PTEN-mTORC2 axis during viral infection. To preferentially interrogate B cell-intrinsic effects within the AID-expressing compartment and minimize potential confounding from pre-GC perturbations (17), we crossed *Pten*^fl/fl^ and *Rictor*^fl/fl^ mice with *Aicda*^Cre^ mice, in which Cre recombinase is expressed under the control of the *Aicda* promoter (33). Using a Cre-reporter system, we confirmed that Cre activity was robustly induced following sheep red blood cells (sRBCs) immunization and was predominantly detected within the GC B cell compartment (**Supplementary Figure S4**). Deletion of PTEN (*Pten*^B^) or both PTEN and Rictor (*Pten*^B^*: Rictor*^B^) in the *Aicda*-expressing B cell population did not alter B cell development or steady-state homeostasis (data not shown). Mice were immunized intraperitoneally with influenza A/WSN virus (IAV) and challenged with a lethal dose of the same strain 21 days post-immunization (**Figure 4A**). All groups exhibited protection and viral clearance following the immunization (**Figures 4B C**), consistent with prior reports (30). Antibody responses to WSN virus were dominated by class-switched isotypes, including IgG1, IgG2b and IgG3 (**Figure 4D**). However, *Pten*^B^ mice showed significantly reduced WSN-specific IgG1 and IgG3 levels, accompanied by a concomitant increase in IgM levels, indicative of defective CSR. Remarkably, mTORC2 inactivation in the PTEN-deficient background (*Pten*^B^*: Rictor*^B^) restored virus-specific IgG1 and IgG3 levels comparable to control mice (**Figure 4D**), highlighting a critical role of mTORC2 in constraining isotype switching during IAV infection.

Although antiviral T cell immunity likely contributed to overall protection in all groups, the CSR defect in *Pten*^B^ mice could compromise antibody-mediated protection. To directly test this, we performed passive immunization by transferring sera from WSN-immunized mice into naïve recipients prior to viral challenge (**Figure 4E**). Sera from immunized control mice conferred robust protection, as evidenced by attenuated weight loss and reduced pulmonary viral loads, whereas sera from *Pten*^B^ mice failed to provide such protection, resembling outcomes in mice that received unimmunized sera (**Figures 4F, G**). Notably, sera from *Pten*^B^*: Rictor*^B^ mice conferred protection equivalent to that of controls immune sera, confirming functional restoration of protective antibody responses by mTORC2 inactivation (**Figures 4F, G**). Histopathological analysis revealed extensive pulmonary inflammation, vascular obstruction, and interstitial hemorrhage in mice that received *Pten*^B^ or unimmunized sera (**Figure 4H**). In contrast, lungs from recipients of control or *Pten*^B^*: Rictor*^B^ sera displayed preserved tissue architecture and reduced inflammation (**Figure 4H**). Together, these results establish that the PTEN-mTORC2 axis critically governs protective, isotype-switched antibody responses against to IAV, and that mTORC2 inhibition can functionally rescue defective humoral immunity caused by PTEN loss.

### PTEN-mTORC2 signaling shapes transcriptional programming in GC-like B cells

**Figure 5:**
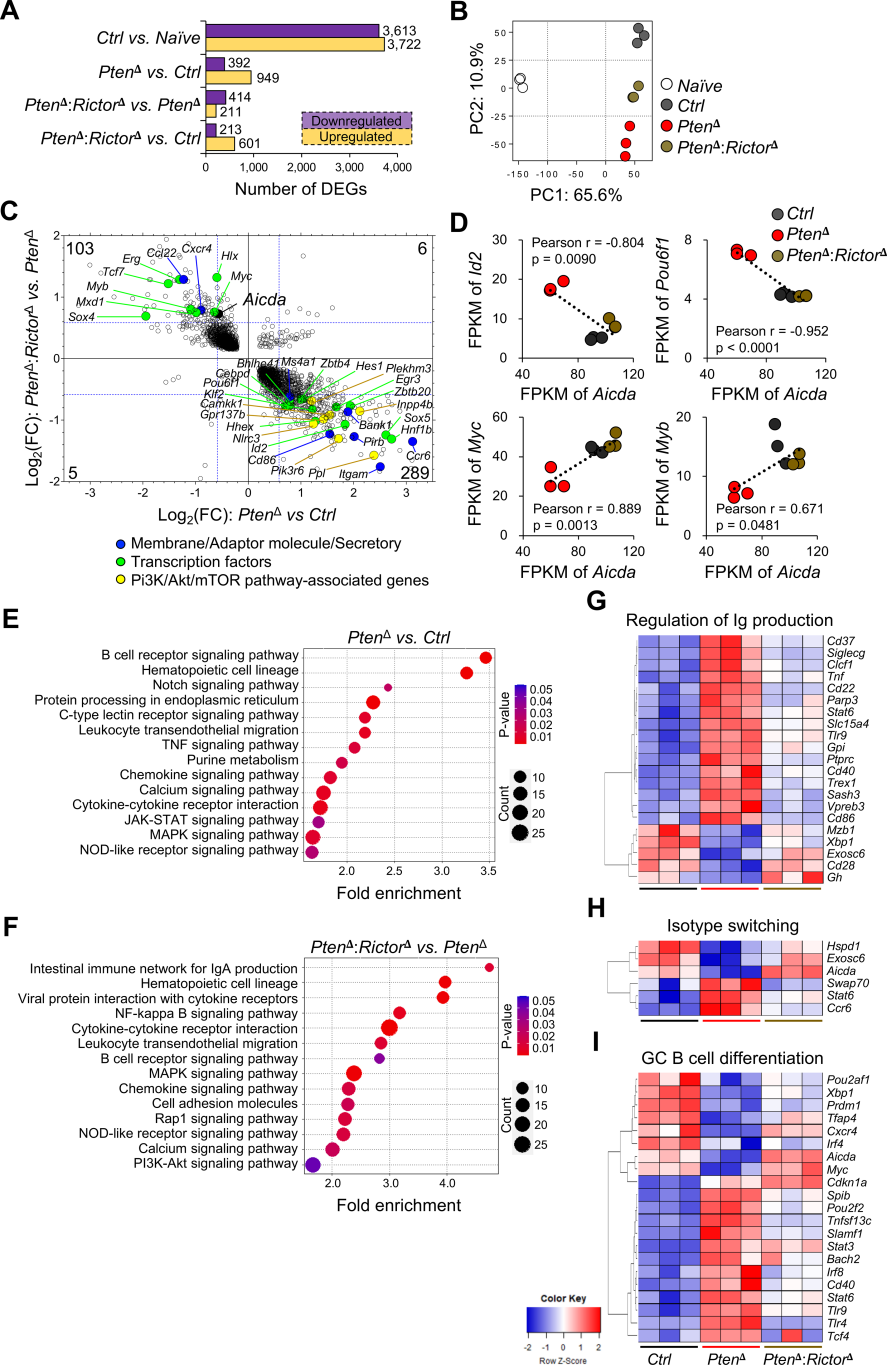
PTEN-mTORC2 axis controls transcriptional programming of iGCBs. iGCBs of the indicated genotypes were cultured and analyzed by RNA-seq. **(A)** Numbers of differentially expressed genes (DEGs) identified in each comparison are shown: n = 3 per group. **(B)** Principal component analysis (PCA) of transcriptomes from naïve B cells and iGCBs. **(C)** Scatter plot showing DEGs reversed by Rictor depletion in PTEN-deficient iGCBs: numbers indicate DEG counts in each quadrant. **(D)** Correlation analyses of *Aicda* expression with *Id2*, *Pou6f1*, *Myc*, or *Myb* expressing levels (FPKM) using Pearson’s correlation analysis: r, Pearson’s correlation coefficient. **(E, F)** Significantly enriched gene sets identified by KEGG pathway analysis in *Pten*^δ^ iGCBs *vs.* control **(E)** and in *Pten*^δ^:*Rictor*^δ^ iGCBs vs. *Pten*^δ^ cells **(F)**. **(G-I)** Heatmaps showing expression of genes associated with immunoglobulin (Ig) production, isotype switching, and GC B cell differentiation pathways.

To define how PTEN-mTORC2 signaling influences GC B cell function at the transcriptional level, we performed RNA-seq analysis on iGCBs. PTEN deficiency led to broad transcriptional perturbation, with 1,341 genes significantly up- or downregulated compared to control cells (**Figure 5A**). Inactivation of mTORC2 in this context resulted in a partial normalization of the transcriptome, with 814 differentially expressed genes (DEGs) identified in *Pten*^δ^:*Rictor*^δ^ iGCBs relative to control. Principle component analysis (PCA) demonstrated clear separation between naïve B cells and iGCBs and further revealed that *Pten*^δ^:*Rictor*^δ^ iGCBs clustered closer to control iGCBs than did *Pten*^δ^ cells (**Figure 5B**). Direct comparison of DEGs revealed that 392 transcripts dysregulated by PTEN loss were reversed upon Rictor deletion, including 289 genes downregulated and 103 genes upregulated (**Figure 5C**). These selectively rescued genes encompassed several regulators relevant to GC identity and isotype switching, including surface receptors (*e.g.*, *Cxcr4* and *Cd86*), transcriptional factors (*e.g.*, *Myc*, *Id2*, *Pou6f1*, and *Myb*), PI3K pathway feedback components (*e.g.*, *Inpp4b* and *Nlrc3*), and *Aicda*, which encodes activation-induced cytidine deaminase (AID). Correlation analyses further showed that expression of *Myc*, *Id2*, *Pou6f1*, and *Myb*, which were positively or negatively associated with *Aicda*, was selectively normalized following mTORC2 inactivation in PTEN-deficient iGCBs (**Figure 5D**). These findings indicate that mTORC2 inhibition restores defined subsets of GC-relevant transcriptional programs disrupted by hyperactive PI3K signaling.

Gene ontology and pathway enrichment analyses of *Pten*^δ^ iGCBs revealed altered representation of multiple signaling pathways, including BCR, MAPK, insulin, and calcium signaling (**Figure 5E**). Importantly, these alterations were partially corrected in *Pten*^δ^:*Rictor*^δ^ iGCBs (**Figures 5E, F**; **Supplementary Figures S5a–d**), supporting a model in which mTORC2 modulates specific transcriptional outputs downstream of PI3K rather than globally resetting GC-associated pathways. Notably, gene sets linked to immunoglobulin (Ig) production and ER function, such as protein processing in ER, were among those most consistently disrupted by PTEN loss and partially restored by mTORC2 inactivation (**Figure 5E**; **Supplementary Figure S5e**), reflecting a potential role of PI3K-mTOR signaling in supporting Ig synthesis and secretory machinery. Consistent with this, expression of *Xbp1*, a transcription factor essential for secretory capacity and plasma cell differentiation, was reduced in *Pten*^δ^ iGCBs and partially restored upon Rictor depletion (**Figure 5G**). Although global enrichment of canonical CSR and GC B cell signature pathways were not observed, heatmap analysis revealed selective normalization of key GC-associated genes, including *Aicda*, *Myc, Cxcr4, Tnfsf13c, Cd40*, and *Bach2* in *Pten*^δ^:*Rictor*^δ^ iGCBs (**Figures 5H, I**). FoxO1, a direct target of Akt that is critical GC zonal organization and *Aicda* regulation (11, 18), was downregulated in PTEN-deficient iGCBs and restored upon mTORC2 inactivation (**Supplementary Figure S5f**). Concordantly, expression of multiple FoxO pathway genes showed partial normalization following mTORC2 inhibition (**Supplementary Figure S5f**). Taken together, these data demonstrate that mTORC2 inhibition selectively rebalances discrete transcriptional programs downstream of hyperactive PI3K signaling, rather than globally restoring GC-associated gene expression. This selective transcriptional rewiring likely underlies the functional rescue of IgG1 class switching and antibody output observed upon mTORC2 inactivation in PTEN-deficient B cells.

### mTORC2 inactivation restores GC-associated B cell differentiation in PTEN-deficient B cells

**Figure 6:**
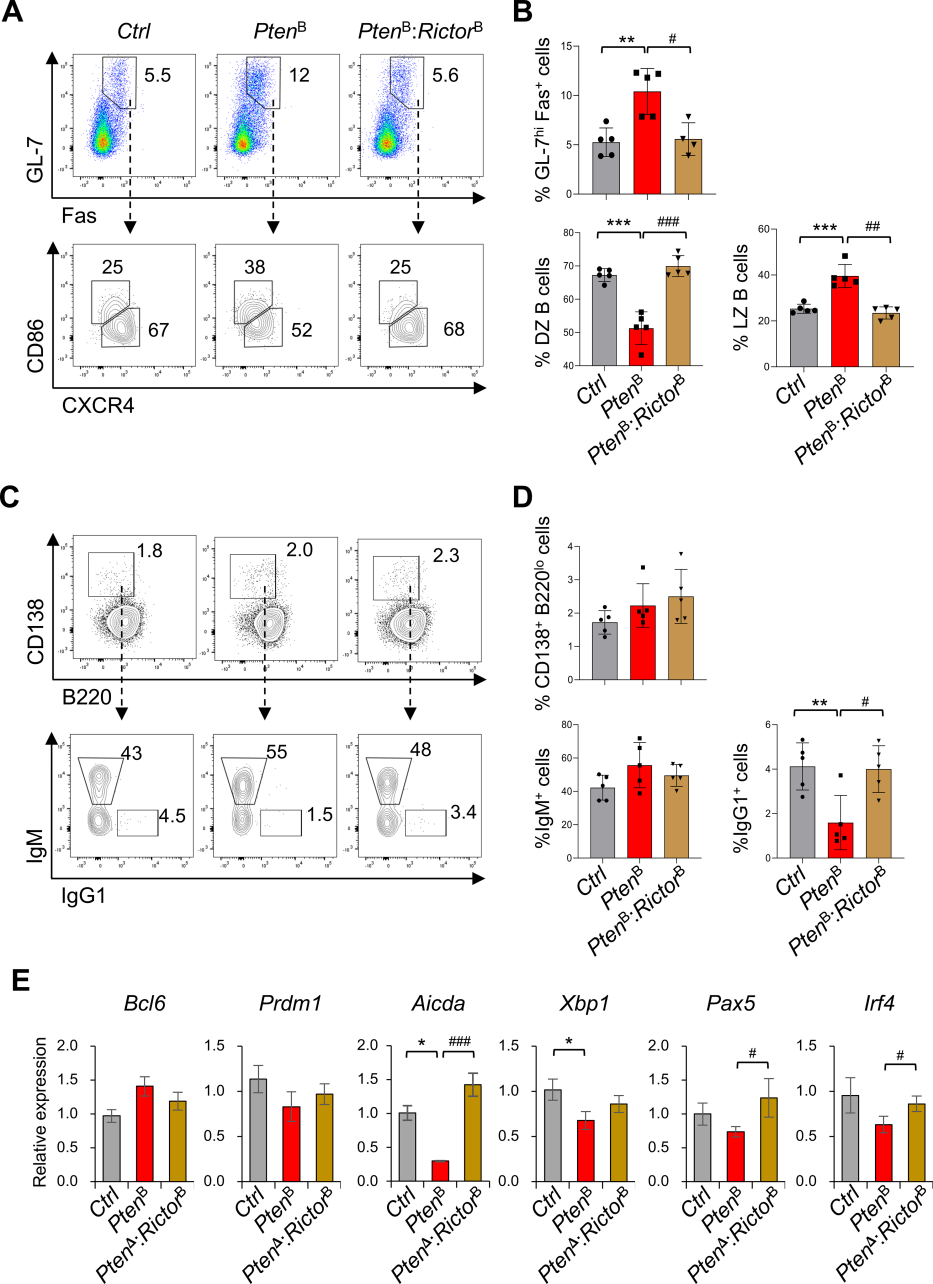
mTORC2 inactivation restores GC-associated B cell differentiation in PTEN-deficient mice. **(A–D)***Aicda*^Cre^-driven conditional KO (*Pten*^B^ and *Pten*^B^*: Rictor*^B^), along with control littermates (*Ctrl*), were immunized with sheep red blood cells (sRBCs), and spleen B cell populations were analyzed by flow cytometry. **(A)** Representative FACS profiles showing GL-7^hi^ Fas^+^ B cells gated on viable B cells, and dark zone (DZ; CXCR4^hi^ CD86^lo^) and light zone (LZ; CXCR4^lo^ CD86^hi^) populations within the GL-7^hi^ Fas^+^ B cell gate. Data shown are representative of two independent experiments, each performed with five or six mice per group. **(B)** Frequencies of total GL-7^hi^ Fas^+^ B cells, DZ cells, and LZ cells are presented as bar graphs: *n* = 5 per group. **(C)** Representative FACS profiles showing CD138^+^ B220^lo^ cells gated on viable B cells and Ig isotype expression within the plasma cell gate. **(D)** Frequencies of total CD138^+^ B220^lo^ cells and those expressing IgM or IgG1 are shown as bar graphs: *n* = 5 per group. **(E)** GL-7^hi^ Fas^+^ B cells were purified from spleens and gene expression was analyzed by quantitative real-time RT-PCR (*n* = 3 per group): *p < 0.05, ** p < 0.01 and *** p < 0.001 compared to *Ctrl*; ^#^p < 0.05, ^##^ p < 0.01 and ^###^ p < 0.001 compared to *Pten*^δ^.

To validate the transcriptional and functional impact of PTEN-mTORC2 signaling *in vivo*, we immunized *Aicda*^Cre^*Pten*^fl/fl^ and *Pten*^fl/fl^:*Rictor*^fl/fl^ mice with sheep red blood cells (sRBCs) and analyzed splenic B cell populations. Consistent with previous finding (13), deletion of PTEN in *Aicda*-expressing B cells resulted in increased frequencies of GL7^hi^ Fas^+^ B cell, a population that includes GC B cells but may also encompass activated extrafollicular *Aicda*-expressing B cells (**Figures 6A, B**; **Supplementary Figures S6a, b**). Notably, *Pten*^B^ mice exhibited an altered distribution of GC-associated subsets, characterized by a reduction in CXCR4^hi^ CD86^lo^ cells and concomitant accumulation of CXCR4^lo^ CD86^hi^ cells, indicative of a skewed DZ-LZ balance and perturbed GC-associated dynamics (**Figures 6A, B**; **Supplementary Figure S6c**). This imbalance was normalized in *Pten*^B^*: Rictor*^B^ mice, implicating mTORC2 in regulating zonal organization and homeostatic differentiation programs within activated *Aicda*-expressing B cells. In agreement with these *in vivo* findings, PTEN-deficient iGCBs displayed reduced *Cxcr4* and elevated *Cd86* expression, features that were reversed upon Rictor deletion (**Figures 5G–I**). Although the overall frequency of CD138^+^ B220^lo^ plasma cells was comparable across groups, the proportion of IgG1^+^ plasma cells was significantly reduced in *Pten*^B^ mice and restored to control levels following mTORC2 inactivation (**Figures 6C, D**). These observations align with our *in vitro* iGCB and iPB differentiation data and support the conclusion that the PTEN-mTORC2 axis negatively regulates IgG1 class switching and downstream antibody-secreting cell output in activated B cells.

To assess transcriptional alterations *in vivo*, we purified splenic GL7^hi^ Fas^+^ B cells from sRBC-immunized mice and analyzed mRNA expression. Transcript levels of *Bcl6* and *Prdm1*, the lineage defining transcriptional factors of GC and plasma cell fate respectively (34), were largely unchanged across genotypes (**Figure 6E**), although *Prdm1* was notably reduced in PTEN-deficient iGCBs (**Figure 5I**). Consistent with RNA-seq data from iGCBs, PTEN deficiency resulted in significant downregulation of *Aicda* and *Xbp1*, both of which are required for CSR and secretory function (35, 36). Importantly, *Aicda* expression was restored to control levels in *Pten*^B^*: Rictor*^B^ cells (**Figure 6E**). While *Pax5* downregulation and *Irf4* induction are associated with GC exit and plasma cell commitment (34), neither transcript was significantly altered in *Pten*^B^ cells. However, both mRNAs were significantly upregulated following mTORC2 inactivation (**Figure 6E**), suggesting that suppression of mTORC2 facilitates transcriptional programs linked to productive plasma cell differentiation. Collectively, these findings support a model in which the PTEN–mTORC2 signaling module integrates transcriptional networks to coordinate differentiation, isotype switching, and effector programming in activated B cells during humoral immune responses.

### PTEN-mTORC2 signaling cross-talks with Notch to regulate isotype switching

**Figure 7:**
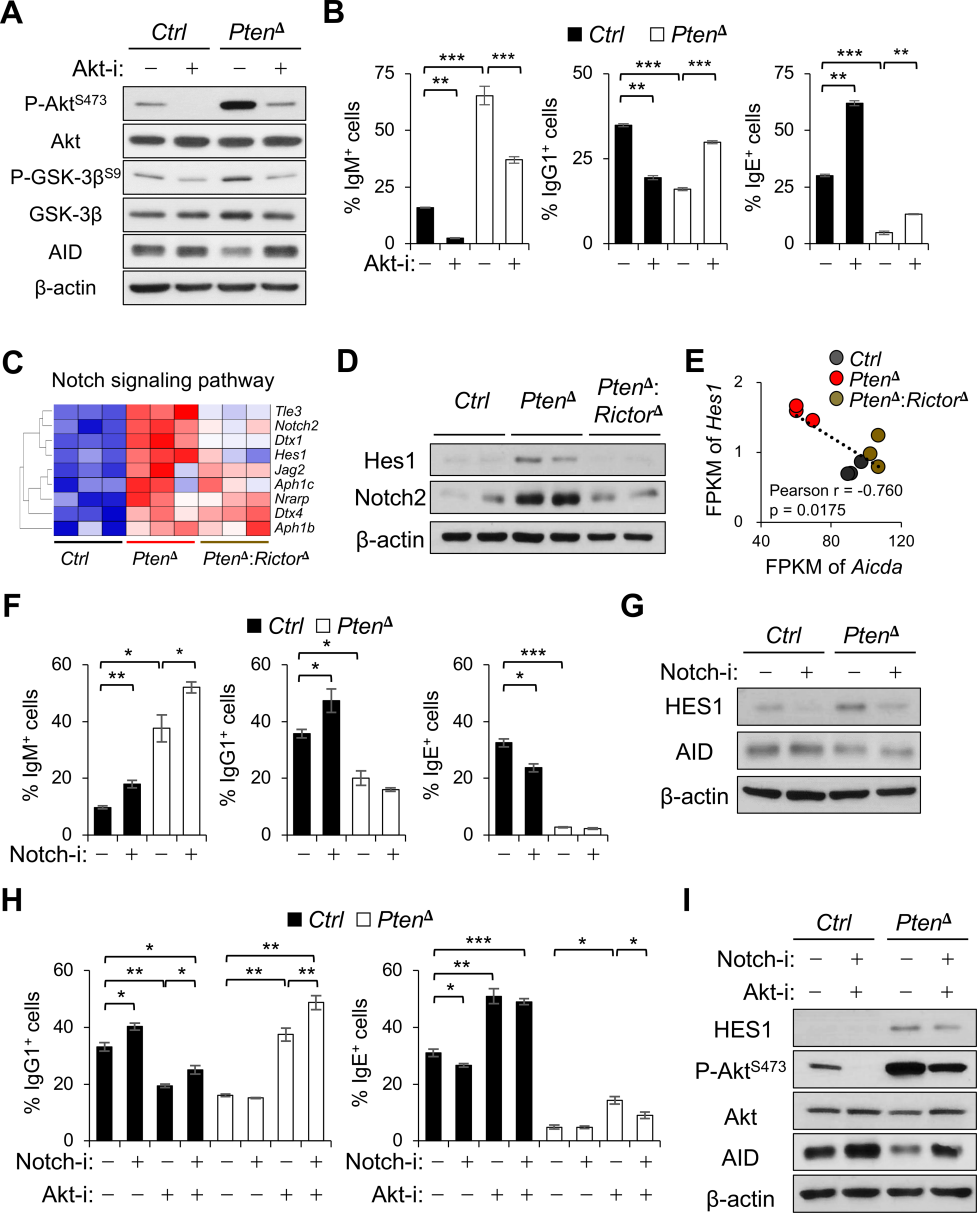
Crosstalk between Akt and Notch signaling pathways regulates IgG1 isotype selection. **(A, B)** iGCBs were cultured in the presence of the Akt inhibitor MK-2206 (Akt-i) or vehicle and analyzed by Western blotting **(A)** and flow cytometry **(B)**. Data shown are representative of three independent experiments, each performed with three biological replicates. **(C)** Expression of genes associated with the Notch signaling pathway is presented as a heatmap, analyzed as in **Figure 5**. **(D)** Protein levels of Hes1 and Notch2 were assessed by Western blotting. Data shown are representative of two independent experiments. **(E)** Negative correlation between *Hes1* and *Aicda* expression levels (FPKM) was determined by Pearson’s correlation analyses: r, Pearson’s correlation coefficient. **(F, G)** iGCBs were cultured in the presence of the Notch inhibitor DAPT (Notch-i), or vehicle and analyzed by flow cytometry **(F)** and immunoblotting **(G)**. Data shown are representative of four independent experiments, each performed with three biological replicates. **(H, I)** iGCBs were cultured with both MK-2206 and DAPT, and analyzed by flow cytometry **(H)** and Western blotting **(I)**. Data shown are representative of three independent experiments, each performed with three biological replicates: *p < 0.05; **p < 0.01; ***p < 0.001.

mTORC2 phosphorylates Akt at S473 within its C-terminal hydrophobic motif, a modification required for full Akt activation and optimal phosphorylation of downstream substrates (17, 37). To interrogate the contribution of Akt activity downstream of mTORC2, we used MK-2206, an allosteric Akt inhibitor that stabilizes Akt in an inactive conformation and thereby prevents both Akt phosphorylation and signaling to downstream targets (38). MK-2206 treatment reduced phosphorylation of Akt as well as that of the Akt substrate GSK-3β, confirming effective inhibition of Akt signaling rather than indirect feedback suppression of mTORC2 activity (**Figure 7A**). Functionally, Akt inhibition restored IgG1 class switching in PTEN-deficient iGCBs (**Figure 7B**). This was accompanied by increased AID expression, whereas IgE levels were only minimally affected (**Figures 7A, B**; **Supplementary Figure S7a**). In contrast, in control iGCBs with intact PTEN, Akt inhibition nearly abolished Akt phosphorylation and resulted in reduced IgG1 switching with a reciprocal increase in IgE expression (**Figures 7A, B**). These data indicate that Akt activity exerts a context-dependent influence on isotype selection, supporting IgG1 switching under a physiological signaling conditions while suppressing CSR when excessively activated downstream of hyperactive PI3K signaling. Consistent with this model, enforced expression of constitutively active Akt impaired IgG1 switching in both control and *Pten*^δ^:*Rictor*^δ^ iGCBs (**Supplementary Figure S7b**), underscoring the requirement for tightly regulated Akt signaling in effective IgG1 switching. Together, these findings support a model in which mTORC2-dependent Akt activity must be precisely tuned: basal Akt signaling is permissive for IgG1 CSR, whereas excessive Akt activation suppresses AID expression and class switching in activated B cells.

The canonical Notch signaling pathway is essential for marginal zone B cell development and has also been implicated in antibody-secreting plasma cell differentiation (39, 40). During *in vitro* GC B cell differentiation, cleavage of intracellular Notch2 (ICN2) was transiently induced and declined during subsequent iPB differentiation (**Supplementary Figure S5c**). Consistent with this observation, transcriptomic analysis revealed enrichment of Notch pathway-associated gene signature in PTEN-deficient iGCBs (**Figure 5E**, **Figure 7C**). Although global Notch pathway enrichment was not observed following mTORC2 inactivation, expression of canonical Notch target genes, including *Hes1* and *Notch2*, was significantly reduced in *Pten*^δ^:*Rictor*^δ^ iGCBs, as confirmed at the protein levels by immunoblotting (**Figures 7C, D**). Notably, *Hes1* expression inversely correlated with *Aicda* transcript levels across genotypes (**Figure 7E**), suggesting that elevated Notch activity might be associated with reduced CSR competence downstream of hyperactive PI3K-mTORC2 signaling.

To explore this relationship functionally, we performed pharmacological perturbation experiments. In control iGCBs, inhibition of Notch cleavage enhanced IgG1 switching and suppressed IgE induction (**Figures 7F, G**). Conversely, enforced expression of the intracellular Notch1 domain (ICN1) modestly but significantly reduced IgG1 switching in both control and *Pten*^δ^:*Rictor*^δ^ iGCBs (**Supplementary Figure S7d**). However, Notch inhibition alone was insufficient to restore IgG1 switching or AID expression in PTEN-deficient iGCBs (**Figures 7F, G**; **Supplementary Figure S7e**), indicating that suppression of Notch cannot overcome the dominant CSR defects imposed by hyperactive PI3K-mTORC2-Akt signaling. Importantly, combined inhibition of Akt and Notch resulted in a significantly greater enhancement of IgG1 switching in PTEN-deficient iGCBs than either perturbation alone (**Figures 7H, I**; **Supplementary Figures S7f, g**). These findings support a model in which Notch signaling does not function as a primary determinant of CSR in this context but instead acts as a cooperating modulatory pathway that can influence isotype selection when PI3K-Akt signaling is partially restrained. Together, our data identify Notch signaling as an auxiliary regulatory input downstream of the PTEN-mTORC2-Akt axis that contributes to fine-tuning antibody isotype outcomes.

## Discussion

CSR and SHM are central processes of GC reactions, regulated by integrated signals from antigen, cytokines, follicular helper T (Tfh) cells, and the stromal microenvironment (1). Among these signals, PI3K signaling is essential for supporting anabolic growth and proliferative expansion of activated B cells. However, excessive PI3K activation paradoxically disrupts GC architecture and impairs isotype switching (10, 11). Although mTOR complexes, key downstream components of the PI3K pathway, have been implicated in CSR regulation (15, 17, 21, 22), an important unresolved question has been how this circuitry operates under pathological PI3K hyperactivation. In this study, we define a PTEN-mTORC2-Akt signaling module that functions within the PI3K network to regulate isotype selection and antiviral humoral immunity. Using conditional genetic models, we demonstrated that mTORC2, rather than mTORC1, dominantly constrains IgG1 switching in PTEN-deficient B cells, and that suppressing mTORC2-Akt activity restores IgG1 CSR without promoting IgE switching. This regulatory module is further validated *in vivo* in both NP-OVA immunization and IAV infection models, where mTORC2 ablation rescues CSR and antibody function in PTEN-deficient mice. Thus, our findings do not redefine the fundamental roles of mTOR complexes in B cells but instead place these activities within a PTEN-PI3K context, revealing how mTORC1 and mTORC2 functions as tunable constraints on isotype selection and humoral immunity.

Our results build upon and refine previous insights into the distinct roles of mTOR complexes during GC responses. For example, *Aicda*^Cre^-driven deletion of Tsc1, an mTORC1 repressor, results in DZ-biased GC architecture and impaired competitive fitness due to defective DZ-to-LZ reentry (16). In contrast, our data demonstrate that under conditions of PTEN deficiency, further inactivation of mTORC1 exacerbates CSR defects, whereas suppression of mTORC2 selectively restores IgG1 switching. These findings underscore the divergent and context-dependent contributions of mTOR complexes to discrete stages of GC B cell differentiation. Spatial segregation of PI3K signaling across GC zones provide an important framework for interpreting these effects. PI3K activity is enriched in LZ B cells, whereas DZ B cells maintain high nuclear FoxO1 levels that are negatively regulated by mTORC2-Akt signaling (11). FoxO1 is required for sustaining the DZ transcriptional program and for AID induction, both of which are compromised upon PTEN deletion or FoxO1 ablation. Consistent with this, PTEN-deficient GC B cells in our study exhibited altered DZ-LZ distribution and reduced expression of key GC fate regulators including *Myc, Irf8* and *Foxo1*. Importantly, these abnormalities were partially corrected by mTORC2 inactivation. Together, these findings support a model in which the PTEN–mTORC2 axis contributes to the maintenance of GC organization and functional competence by modulating, rather than fully reprogramming, zonal and transcriptional states.

Our transcriptomic analyses further reinforce the concept that the PTEN-mTORC2-Akt module shapes selective transcriptional programs that couple PI3K signaling intensity to GC-associated gene networks. Inactivation of mTORC2 did not globally normalize the PTEN-deficient transcriptome but instead partially restored defined subsets of genes linked to GC organization (*e.g.*, *Cxcr4, Cd86*), signaling feedback loops (*e.g.*, *Inpp4b, Nlrc3*), and Ig synthesis machinery (*e.g.*, *Xbp1*). Although Myc is required for AP4-dependent proliferation of DZ B cells following LZ section (41), it is insufficient on its own to sustain robust CSR under conditions of excessive PI3K activity (42). Accordingly, the rescue of IgG1 switching observed upon mTORC2 inhibition likely reflects broader transcriptional rebalancing across multiple GC-relevant pathways rather than correction of a single regulatory node. This selective transcriptional modulation is particularly evident during the GC-to-plasma cell transition (34): Hyperactivated PI3K signaling suppressed the expression of *Irf4*, *Pax5*, *Xbp1*, and genes involved in Ig synthesis and processing, whereas mTORC2 inhibition partially reinstates their expression in both iGCBs and *in vivo* AID-expressing B cell populations. Collectively, these data indicate that targeted attenuation of mTORC2 activity enables late-stage GC maturation and productive antibody output by reprogramming of key functional modules.

We further demonstrate that selective disruption of mTORC2 rescues functional defects caused by PTEN loss *in vivo*. PTEN-deficient B cells failed to mount effective IgG1 responses after immunization, but these defects were corrected by mTORC2 ablation. Notably, Akt inhibition enhanced IgG1 switching in PTEN-deficient cells but increased IgE production in wild-type iGCBs, revealing an activity-dependent role of mTORC2-Akt signaling in isotype selection. Restoration of NP-specific IgG3 and IgA responses, together with recovery of IgG3 responses following IAV infection supports broader roles for the PTEN-mTORC2 module in regulating CSR. Interestingly, IgG2b responses were enhanced by mTORC2 inactivation in the NP-immunization model but not during IAV infection, suggesting context-dependent regulation that may be influenced by distinct cytokine and antigen receptor inputs (43). Among cytokine receptors, *Tnfsf13c*, encoding BAFF-R, was markedly upregulated in PTEN-deficient iGCBs and normalized upon mTORC2 inactivation (**Figure 5I**). Because BAFF-R signaling can enhance AID expression via the non-canonical NF-κB signaling (43), further studies will be required to determine whether this pathway contributes to PTEN-mTORC2-mediated regulation of CSR.

Using a passive immunization model, we further demonstrate that suppression of mTORC2 restores the protective capacity of antibodies derived from PTEN-deficient B cells against IAV infection. These findings align with, yet also extends, prior reports that pharmacologic mTOR inhibition can enhance heterotypic influenza immunity by reshaping antibody repertoires during vaccination, albeit often at the expense of GC integrity and class switching (20). Likewise, mice harboring a hypomorphic or null mTOR alleles fail to mount effective protection against *S. pneumoniae* infection, due to GC collapse or defective plasma cell formation (21). In contrast, selective disruption of mTORC2 in the context of PTEN deficiency preserves GC output while restoring isotype switching, highlighting a critical distinction between broad mTOR inhibition and targeted modulation of PTEN-mTORC2-Akt axis. Although our NP-based immunization data indicate enhanced binding to NP_2_ and cross-reactive binding to NIP, suggestive of increase antibody avidity in *Pten*^B^*: Rictor*^B^ mice, we note that the influenza protection experiments do not directly resolve whether improved viral control is driven increased affinity, altered epitope specificity or enhanced Fc-mediated effector function. Given that IgG isotypes exhibited superior neutralization, complement activation, and Fc receptor engagement compared to IgM (2, 3), an increased IgG-to-IgM ratio alone could substantially enhance antiviral immunity. Future studies incorporating antibody repertoire sequencing and somatic hypermutation profiling will be required to determine how mTORC2 modulation influences affinity maturation and clonal selection during viral infection. Together, these observations underscore the context-dependent mTORC2 in humoral immunity and supports the concept that selective tuning of the PTEN-mTORC2-Akt signaling module, rather than global mTOR inhibition, represents a more precise strategy to optimize antibody quality during infection or vaccination.

An additional novel aspect of our study is the identification of a functional association between mTORC2-Akt signaling and the Notch pathway in shaping isotype outcomes. Canonical Notch signaling is well established in marginal zone B cell development and has also been implicated in plasma cell differentiation (39, 40). Emerging evidence indicates that disruption of Notch signaling compromises protective immunity to influenza infection, and genetic variations in the Notch pathway have been associated with increased COVID-19 susceptibility in aged populations (44, 45). In this context, we observed that PTEN-deficient iGCBs exhibited elevated expression of canonical Notch target genes, including *Hes1* and *Notch2*, and that this increase was attenuated by concurrent mTORC2 inactivation. Functionally, pharmacological inhibition of Notch alone was insufficient to restore AID expression or IgG1 switching in PTEN-deficient iGCBs, indicating that Notch activity is unlikely to be the primary driver of the CSR defect imposed by hyperactive PI3K signaling. Nonetheless, Notch blockade promoted IgG1 switching and suppressed IgE in wild-type cells, effects distinct from those observed following Akt inhibition. Importantly, Notch inhibition augmented the capacity of Akt blockade to restore IgG1 switching in PTEN-deficient iGCBs, suggesting that Notch signaling can modulate isotype selection when PI3K-Akt signaling is partially restrained (46). These observations expand upon previous studies showing that PTEN loss or hyperactivation of p110α suppresses IgG1 CSR through Akt-dependent mechanisms, whereas IgE switching appears to involve additional, Akt-independent regulatory pathways (12, 21). Although definitive assessment of Notch pathway necessity will require GC-specific genetic perturbation of canonical Notch components, our data support a model in which Notch signaling functions as an auxiliary regulatory input within the PI3K network, contributing to fine-tuning of isotype selection rather than acting as a dominant determinant of CSR.

Our findings provide insight into the paradoxical humoral immunodeficiency observed in patients with either activating or inactivating in *PI3KCD* mutations, both of which impair CSR and Ig production (16, 47). Rather than reflecting a simple gain- or loss-of-function phenotype, these clinical observations are consistent with a requirement for precise calibration of PI3K signaling strength during B cell activation. We propose that dysregulation of the PTEN-mTORC2-Akt module contributes to this phenotype by disrupting integration of upstream activation cues, potentially through sustained Akt activity that suppresses transcriptional programs required for AID induction and productive class switching (48).

Importantly, prior work has demonstrated that PTEN regulates GC responses by remodeling of signaling downstream of multiple inputs, including cytokine receptors, in addition to antigen receptor and CD40 (23). In line with this, our data suggest that PTEN-mTORC2-Akt regulation represents a general tuning mechanism within the PI3K network. This interpretation is supported by our observation that mTORC2 inactivation restored IL-4-dependent IgG1 switching in PTEN-deficient B cells activated through both CD40-dependnet GC-like cultures and LPS-driven, T-independent plasmablast cultures (**Supplementary Figure S2h**). Thus, the PTEN-mTORC2 axis appears to calibrate downstream transcriptional and metabolic program across distinct contexts in which PI3K is engaged. While our *in vitro* GC-like culture system recapitulates many aspects of GC biology, it does not incorporate antigen-dependent BCR selection or the full complexity of the Tfh-GC niche. Accordingly, conclusions regarding GC physiology are supported primarily by complementary *in vivo* genetic data. Moreover, *Aicda*^Cre^ targets a broader AID-expressing B cell compartment that includes both extrafollicular and GC B cells, and antibody readouts therefore integrate contributions from multiple phases of humoral response. Further studies employing lineage-trancing approaches and recall immunization models will be required to define how this signaling module regulates memory B cell formation and recall antibody response across dynamic GC microenvironments.

In summary, we define the PTEN-mTORC2-Akt signaling module as a key regulatory circuit governing activated B cell differentiation, antibody isotyping selection, and antiviral humoral immunity. Functional interaction with the Notch pathway adds an additional layer of modulation to antibody diversification. Together, our findings provide a framework for understanding how PI3K signal strength is translated into qualitative antibody outputs and suggest that selective modulation of this signaling axis could be harnessed to enhance vaccine-induced immunity or limit antibody-driven immunopathology in infectious and autoimmune diseases.

## Data Availability

The datasets presented in this study can be found in online repositories. The names of the repository/repositories and accession number(s) can be found below: https://www.ncbi.nlm.nih.gov/, GSE298707.

## Supplementary material

Supplementary Table 1 Primer sequences used in quantitative real-time PCR analysis.

Supplementary Figure 1 Effect of mTOR inhibitors on GC-like B cell activation markers. B cells were cocultured with CD40LB feeder cells in the presence of IL-4 to generate in vitro GC-like B cells (iGCBs), followed by IL-21-driven differentiation into in vitro plasmablasts (iPBs), as described in **Figure 1A**. **(a)** Quantification of the time course immunoblot analysis shown in **Figure 1B**. Relative band intensities of P-S5, P-4E-BP1, and P-Akt were normalized to their corresponding total protein levels. **(b)** Fold increase in viable cell numbers relative to the input cells four days of iGCB culture. **(c)** Quantification of immunoblot analysis of iGCBs cultured in the presence of rapamycin (Rapa), torin 2 (Torin), or vehicle (Vh), corresponding to the data shown in **Figure 1C**. **(d)** iGCBs cultured in the presence of Rapa, Torin, or Vh were analyzed by flow cytometry, as described in **Figure 1**. Representative histograms gated on viable B cells are shown, with mean fluorescence intensity (MFI) values of the indicated markers.

Supplementary Figure 2 Inactivation of mTORC2 enhances IgG1 isotype switching in PTEN-deficient iGCBs. **(a)** Fold increase in viable cell numbers relative to the input cells four days of iGCB culture. **(b)** Quantification of the immunoblot analysis shown in **Figure 2B**. Relative band intensities of P-S6 and P-Akt were normalized to their respective total protein levels. **(c, d)** Intracellular PIP3 levels in naïve B cells **(c)** and iGCBs (d) from PtenD, PtenD:RaptorD, PtenD:RictorD, and control mice were measured by flow cytometry using an anti-PIP3 antibody. **(e)** Representative FACS histograms gated on viable B cells, showing MFI of the indicated B cell activation markers. **(f)** Four days after iPB differentiation, relative amounts of Ig isotypes in culture supernatants were determined by ELISA (n = 4): *, p < 0.05, ** p < 0.01 and ***, p < 0.001 compared to Ctrl; ##, p < 0.01 and ###, p < 0.001 compared to PtenD. **(g)** iGCBs were cultured and germline Iµ-Cg1 transcript levels were measured by quantitative real-time PCR. Expression levels were normalized to Actb. **(h)** B cells were activated with LPS (5 µg/ml) and BAFF (50 ng/ml) in the presence or absence of IL-4 (10 ng/ml) for four days and 3 analyzed by flow cytometry. Representative FACS plots gated on viable B cells are shown, with percentages of IgG1+ cells indicated.

Supplementary Figure 3 The PTEN-mTORC2 axis regulates antibody class switching in vivo. Rag2 /- mice were reconstituted with B cells of the indicated genotypes together with wild-type CD4+ T cells and immunized with NP-OVA, as described in **Figure 3**. Five days after booster immunization, splenic B cell populations were analyzed by flow cytometry. **(a, b)** Representative gating strategies used to identify GL-7hi Fas+ B cells **(a)** and CD138+ B220lo plasma cells **(b)**. GL-7hi Fas+ B cells were defined as CD19+ B220+ IgD1- GL-7hi Fas+ cells, and plasma cells were defined as CD19+ IgD- B220lo CD138+ cells. (c, d) Absolute numbers of GC B cells **(c)** and plasma cells **(d)** in the spleen of the indicated mice.

Supplementary Figure 4 Cellular distribution of Cre activity in AicdaCre mice. AicdaCre mice were crossed with ROSA26LSL-YFP reporter mice, and immunized intraperitoneally with sheep red blood cells (sRBC). Splenic B cell populations were analyzed by flow cytometry at day 5 and 10 post immunization, as described in **Figure 4**. **(a)** Representative FACS profiles showing YFP expression in CD19+ B cells and non-B cell populations with percentages of YFP+ cells, respectively. **(b)** FACS plots and proportions of YFP+ cells in GL-7hi CD95+ B cells.

Supplementary Figure 5 PTEN-mTORC2 axis controls transcriptional programming of iGCBs. **(a–f)** Heatmaps for BCR signaling, MAPK signaling, insulin signaling, calcium signaling, protein processing in ER and FoxO signaling pathways are shown.

Supplementary Figure 6 mTORC2 inactivation restores GC-associated B cell differentiation in PTEN deficient mice. AicdaCre-driven conditional KO mice were immunized with sRBCs, and splenic B cell populations were analyzed by flow cytometry, as described in **Figure 6**. **(a, b)** Representative gating 4 strategies used to identify GL-7hi Fas+ B cells **(a)** and plasma cells **(b)**. Fas+ GL-7hi B cells were defined as CD45+ CD19+ IgD- Fas+ GL-7hi cells, and plasma cells were defined as CD45+ CD19+ IgD- B220lo CD138+ cells. **(c)** Representative FACS histograms gated on viable B cells, showing MFI of CD86.

Supplementary Figure 7 Crosstalk between Akt and Notch signaling pathways regulates IgG1 selection. **(a)** iGCBs were cultured in the presence of the Akt inhibitor MK-2206 (Akt-i) or vehicle and analyzed by Western blotting, as shown in **Figure 7**. Quantification of AID protein expression from the immunoblots shown in **Figure 7A**. Relative band intensities were normalized to b-actin. **(b)** iGCBs were retrovirally transduced with constitutively active Akt (Myr-Akt) or control vector (MiG), and IgG1 switching was assessed by flow cytometry. **(c)** iGCBs and iPBs were cultured for the indicated times and cleavage of the Notch2 intracellular domain (ICN2) was analyzed by Western blotting. **(d)** iGCBs were retrovirally transduced with intracellular Notch1 (ICN1) or control vector (MiG), and IgG1 switching was analyzed by flow cytometry. (e-g) iGCBs were cultured in the presence of MK-2206 (Akt-i) and/or Notch inhibitor DAPT (Notch-i) as in **Figure 7**. **(e, f)** Quantification of AID protein expression from the immunoblot in **Figure 7G, I**, respectively. (g) Ratio of IgG1+ to IgE+ iGCBs.
