# Supplementary material for: PTEN-mTORC2 signaling module controls antibody isotype selection and antiviral humoral immunity
Source: Front Immunol. 2026 Feb 10;17:1771230. doi: 10.3389/fimmu.2026.1771230 (PMC12929395; doi:10.3389/fimmu.2026.1771230)

SUPPLEMENTAL INFORMATION

PTEN-mTORC2 signaling module controls antibody isotype selection and antiviral humoral immunity

Bikash Thapa[1†‡], Yejin Lee[2‡], Seongwon Pak[2], Dongkyu Kim[2], Hyung-Joo Kwon[3] and Keunwook Lee[1, 2*]

[1]Institute of Bioscience & Biotechnology, Hallym University, Chuncheon, Republic of Korea.

[2]Department of Biomedical Science, Hallym University, Chuncheon, Republic of Korea.

[3]Department of Microbiology, College of Medicine, Hallym University, Chuncheon, Republic of Korea.

*Correspondence: Keunwook Lee

Email. keunwook@hallym.ac.kr

†Present address: Center for Immunology, Fox Chase Cancer Center, Philadelphia PA, United States

‡These authors have contributed equally to this work

Supplemental Table S1. Primer sequences used in quantitative real-time PCR analysis.

Gene name	Sequence (5' to 3')
Actb	Forward: GGCACCACACCTTCTACAATG<br>Reverse: GGGGTGTTGAAGGTCTCAAAC
influenza A virus nucleocapsid protein (NP)	Forward: TGA GAG AGA GCC GGA ATC CA<br>Reverse: ATC CAT ACA CAC AGG CAG GC
Bcl6	Forward: GGA AAG GCC GGA CAC CAG TT<br>Reverse: CCG GAG GCG ATT AAG GTT GA
Prdm1	Forward: GCC AAC CAG GAA CTT CTT GTG T<br>Reverse: AGG ATA AAC CAC CCG AGG GT
Aicda	Forward: GGC ATG AGA CCT ACC TCT GC<br>Reverse: CAG GAG GTG AAC CAG GTG AC
Xbp1	Forward: AAC ACG CTT GGG AAT GGA CA<br>Reverse: ACA TAG TCT GAG TGC TGC GG
Pax5	Forward: TCT ACA GGC TCC GTG ACG CA<br>Reverse: GAA CAG GTC TCC CCG CAT CT
Irf4	Forward: CTT TGA GGA ATT GGT CGA GAG G<br>Reverse: GAG AGC CAT AAG GTG CTG TCA

Supplemental Figure Legends

Supplemental Figure S1. Effect of mTOR inhibitors on GC-like B cell activation markers. B cells were cocultured with CD40LB feeder cells in the presence of IL-4 to generate *in vitro* GC-like B cells (iGCBs), followed by IL-21-driven differentiation into *in vitro* plasmablasts (iPBs), as described in Figure 1A. (a) Quantification of the time course immunoblot analysis shown in Figure 1B. Relative band intensities of P-S5, P-4E-BP1, and P-Akt were normalized to their corresponding total protein levels. (b) Fold increase in viable cell numbers relative to the input cells four days of iGCB culture. (c) Quantification of immunoblot analysis of iGCBs cultured in the presence of rapamycin (Rapa), torin-2 (Torin), or vehicle (Vh), corresponding to the data shown in Figure 1C. (d) iGCBs cultured in the presence of Rapa, Torin, or Vh were analyzed by flow cytometry, as described in Figure 1. Representative histograms gated on viable B cells are shown, with mean fluorescence intensity (MFI) values of the indicated markers.

Supplemental Figure S2. Inactivation of mTORC2 enhances IgG1 isotype switching in PTEN-deficient iGCBs. (a) Fold increase in viable cell numbers relative to the input cells four days of iGCB culture. (b) Quantification of the immunoblot analysis shown in Figure 2B. Relative band intensities of P-S6 and P-Akt were normalized to their respective total protein levels. (c, d) Intracellular PIP_3 levels in naïve B cells (c) and iGCBs (d) from *Pten*$^{\Delta}$, *Pten*$^{\Delta}$:*Raptor*$^{\Delta}$, *Pten*$^{\Delta}$:*Rictor*$^{\Delta}$, and control mice were measured by flow cytometry using an anti-PIP_3 antibody. (e) Representative FACS histograms gated on viable B cells, showing MFI of the indicated B cell activation markers. (f) Four days after iPB differentiation, relative amounts of Ig isotypes in culture supernatants were determined by ELISA (n = 4): *, $p < 0.05$, ** $p < 0.01$ and ***, $p < 0.001$ compared to *Ctrl*; $^{\#\#}$, $p < 0.01$ and $^{\#\#\#}$, $p < 0.001$ compared to *Pten*$^{\Delta}$. (g) iGCBs were cultured and germline Iμ-Cγ1 transcript levels were measured by quantitative real-time PCR. Expression levels were normalized to *Actb*. (h) B cells were activated with LPS (5 μg/ml) and BAFF (50 ng/ml) in the presence or absence of IL-4 (10 ng/ml) for four days and analyzed by flow cytometry. Representative FACS plots gated on viable B cells are shown, with

percentages of IgG1$^+$ cells indicated.

Supplemental Figure S3. The PTEN-mTORC2 axis regulates antibody class switching *in vivo*. *Rag2*$^{-/-}$ mice were reconstituted with B cells of the indicated genotypes together with wild-type CD4$^+$ T cells and immunized with NP-OVA, as described in Figure 3. Five days after booster immunization, splenic B cell populations were analyzed by flow cytometry. (a, b) Representative gating strategies used to identify GL-7^{hi} Fas$^+$ B cells (a) and CD138$^+$ B220^{lo} plasma cells (b). GL-7^{hi} Fas$^+$ B cells were defined as CD19$^+$ B220$^+$ IgD1$^-$ GL-7^{hi} Fas$^+$ cells, and plasma cells were defined as CD19$^+$ IgD$^-$ B220^{lo} CD138$^+$ cells. (c, d) Absolute numbers of GC B cells (c) and plasma cells (d) in the spleen of the indicated mice.

Supplemental Figure S4. Cellular distribution of Cre activity in *Aicda*^{Cre} mice. *Aicda*^{Cre} mice were crossed with *ROSA26*$^{LSL-YFP}$ reporter mice, and immunized intraperitoneally with sheep red blood cells (sRBC). Splenic B cell populations were analyzed by flow cytometry at day 5 and 10 post-immunization, as described in Figure 4. (a) Representative FACS profiles showing YFP expression in CD19$^+$ B cells and non-B cell populations with percentages of YFP$^+$ cells, respectively. (b) FACS plots and proportions of YFP$^+$ cells in GL-7^{hi} CD95$^+$ B cells.

Supplemental Figure S5. PTEN-mTORC2 axis controls transcriptional programming of iGCBs. (a-f) Heatmaps for BCR signaling, MAPK signaling, insulin signaling, calcium signaling, protein processing in ER and FoxO signaling pathways are shown.

Supplemental Figure S6. mTORC2 inactivation restores GC-associated B cell differentiation in PTEN-deficient mice. *Aicda*^{Cre}-driven conditional KO mice were immunized with sRBCs, and splenic B cell populations were analyzed by flow cytometry, as described in Figure 6. (a, b) Representative gating strategies used to identify GL-7^{hi} Fas$^+$ B cells (a) and plasma cells (b). Fas$^+$ GL-7^{hi} B cells were defined as CD45$^+$ CD19$^+$ IgD$^-$ Fas$^+$ GL-7^{hi} cells, and plasma cells were defined as CD45$^+$ CD19$^+$ IgD$^-$ B220^{lo}

$CD138^+$ cells. (c) Representative FACS histograms gated on viable B cells, showing MFI of CD86.

Supplemental Figure S7. Crosstalk between Akt and Notch signaling pathways regulates IgG1 selection. (a) iGCBs were cultured in the presence of the Akt inhibitor MK-2206 (Akt-i) or vehicle and analyzed by Western blotting, as shown in Figure 7. Quantification of AID protein expression from the immunoblots shown in Figure 7A. Relative band intensities were normalized to β-actin. (b) iGCBs were retrovirally transduced with constitutively active Akt (Myr-Akt) or control vector (MiG), and IgG1 switching was assessed by flow cytometry. (c) iGCBs and iPBs were cultured for the indicated times and cleavage of the Notch2 intracellular domain (ICN2) was analyzed by Western blotting. (d) iGCBs were retrovirally transduced with intracellular Notch1 (ICN1) or control vector (MiG), and IgG1 switching was analyzed by flow cytometry. (e-g) iGCBs were cultured in the presence of MK-2206 (Akt-i) and/or Notch inhibitor DAPT (Notch-i) as in Figure 7. (e, f) Quantification of AID protein expression from the immunoblot in Figure 7G and I, respectively. (g) Ratio of $IgG1^+$ to IgE^+ iGCBs.

Thapa and Lee *et al.* Supplemental Figure S1

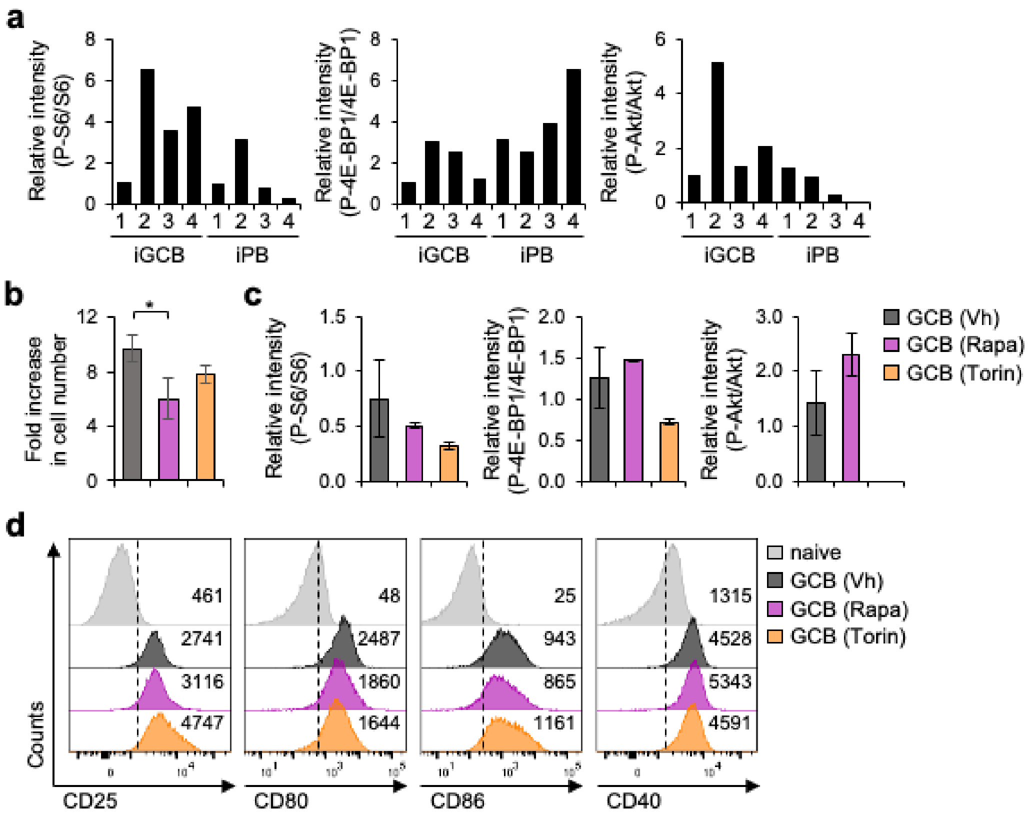

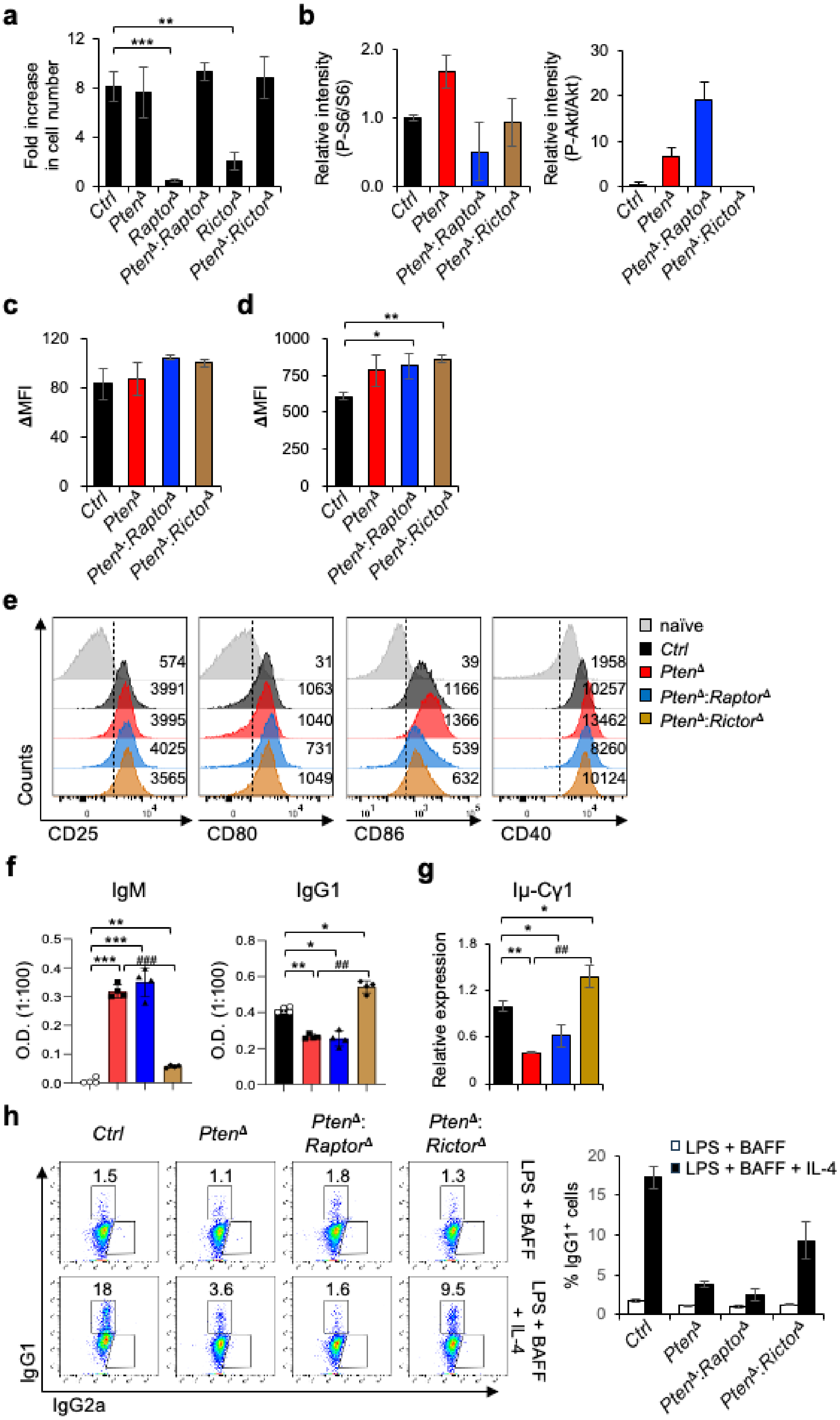

a
Fold increase in cell number
Ctrl
PtenΔ
RaptorΔ
PtenΔ:RaptorΔ
RictorΔ
PtenΔ:RictorΔ
b
Relative intensity (P-S6/S6)
Relative intensity (P-Akt/Akt)
c
ΔMFI
d
e
naïve
Counts
CD25
CD80
CD86
CD40
f
IgM
IgG1
O.D. (1:100)
g
Iμ-Cγ1
Relative expression
h
IgG1
IgG2a
LPS + BAFF
LPS + BAFF + IL-4
% IgG1+ cells

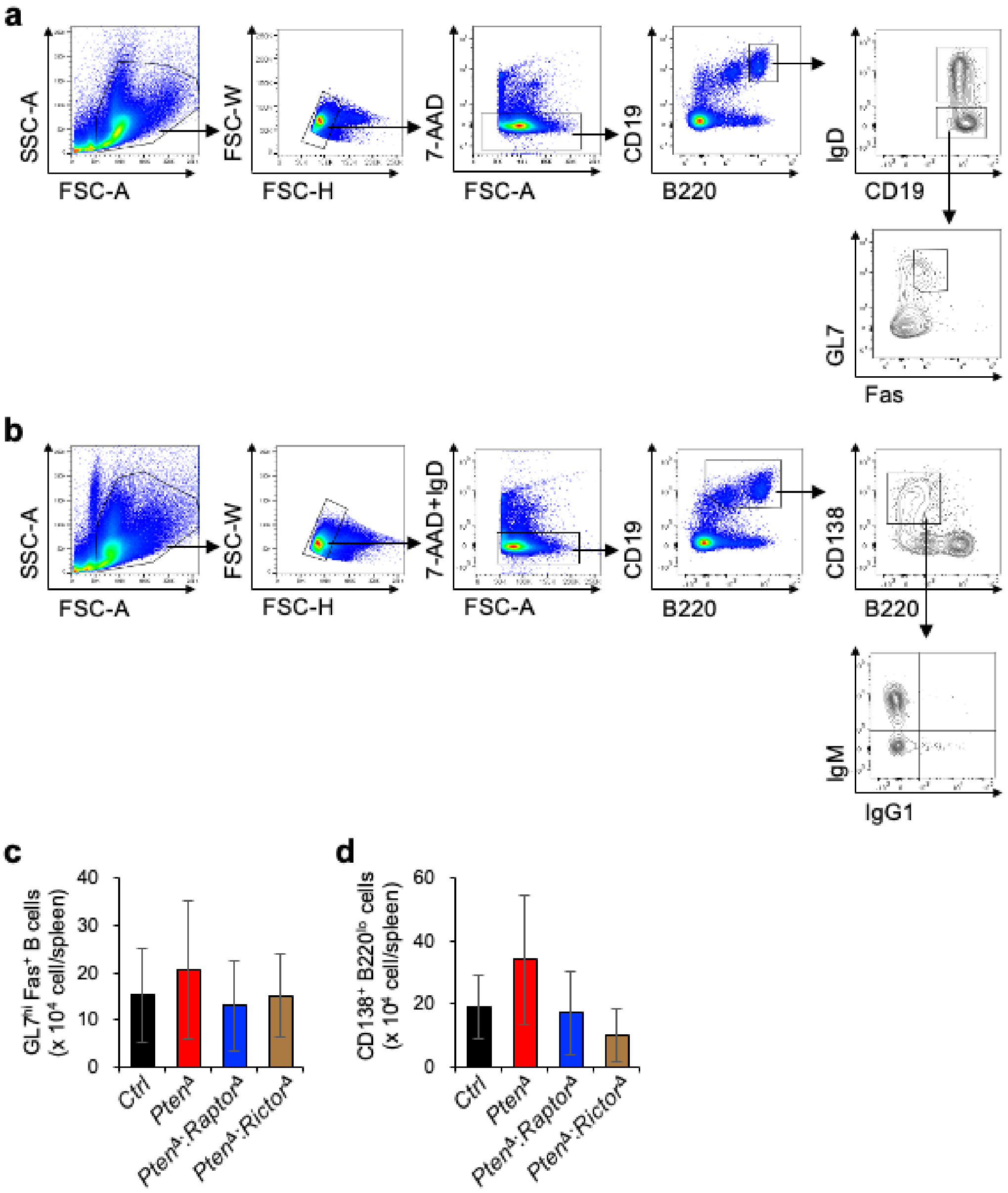

a
SSC-A
FSC-A
FSC-W
FSC-H
7-AAD
FSC-A
CD19
B220
IgD
CD19
GL7
Fas
b
SSC-A
FSC-A
FSC-W
FSC-H
7-AAD+IgD
FSC-A
CD19
B220
CD138
B220
IgM
IgG1
c
GL7hi Fas+ B cells
(x 10⁴ cell/spleen)
40
30
20
10
0
Ctrl
PtenΔ
PtenΔ-RaptorΔ
PtenΔ-RictorΔ
d
CD138+ B220lo cells
(x 10⁴ cell/spleen)
60
40
20
0
Ctrl
PtenΔ
PtenΔ-RaptorΔ
PtenΔ-RictorΔ

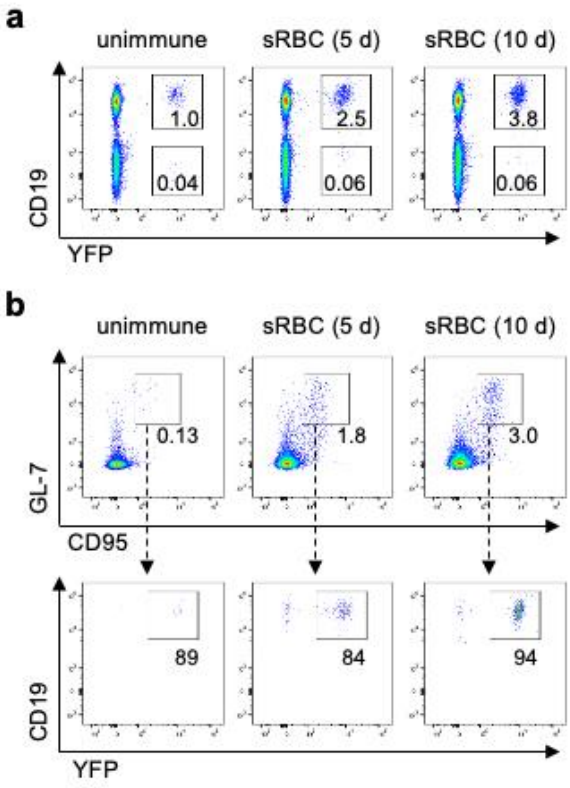
a
unimmune
sRBC (5 d)
sRBC (10 d)
1.0
0.04
2.5
0.06
3.8
0.06
CD19
YFP
b
unimmune
sRBC (5 d)
sRBC (10 d)
0.13
1.8
3.0
GL-7
CD95
89
84
94
CD19
YFP

Thapa and Lee *et al.* Supplemental Figure S5

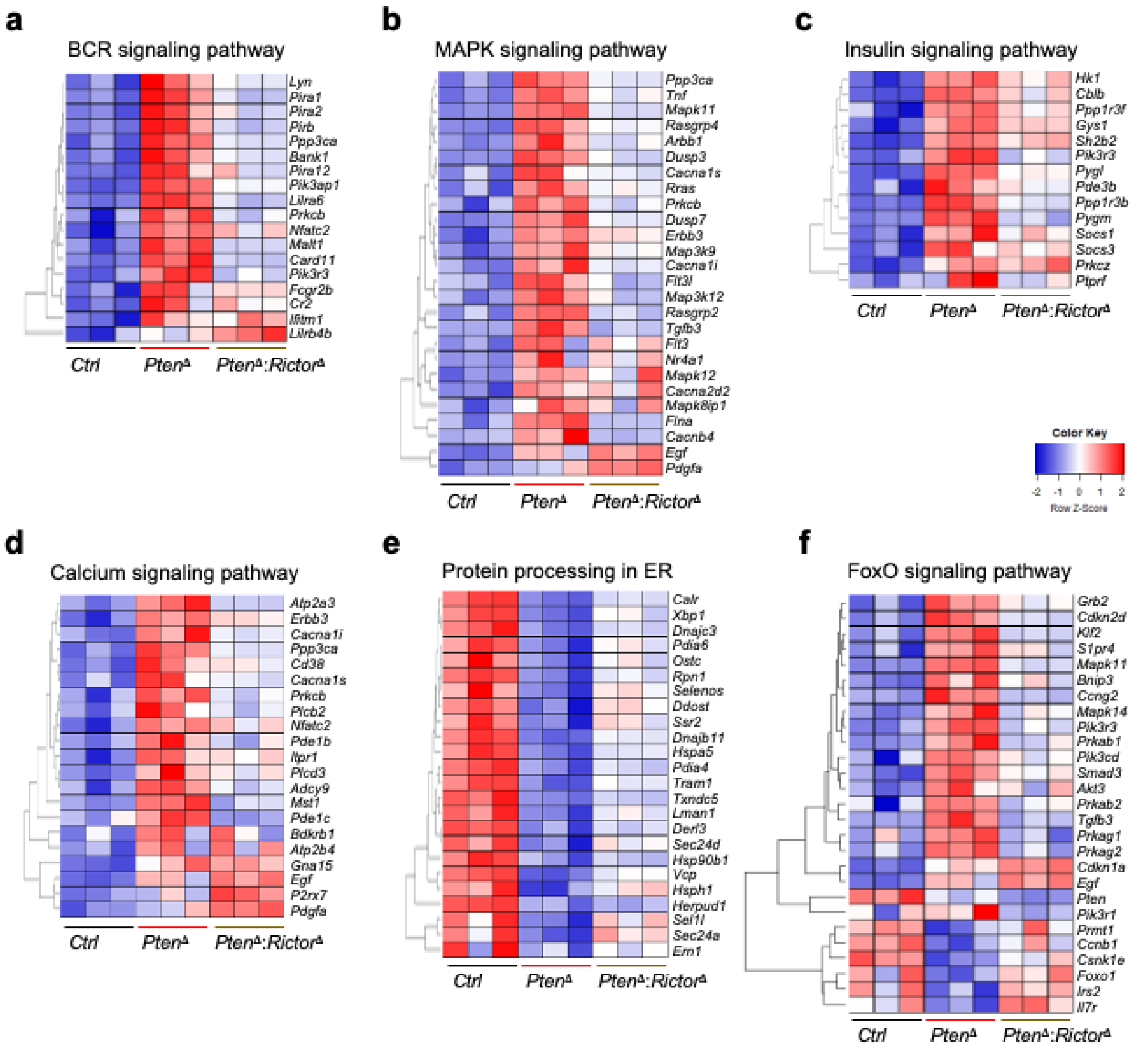

Thapa and Lee *et al.* Supplemental Figure S6

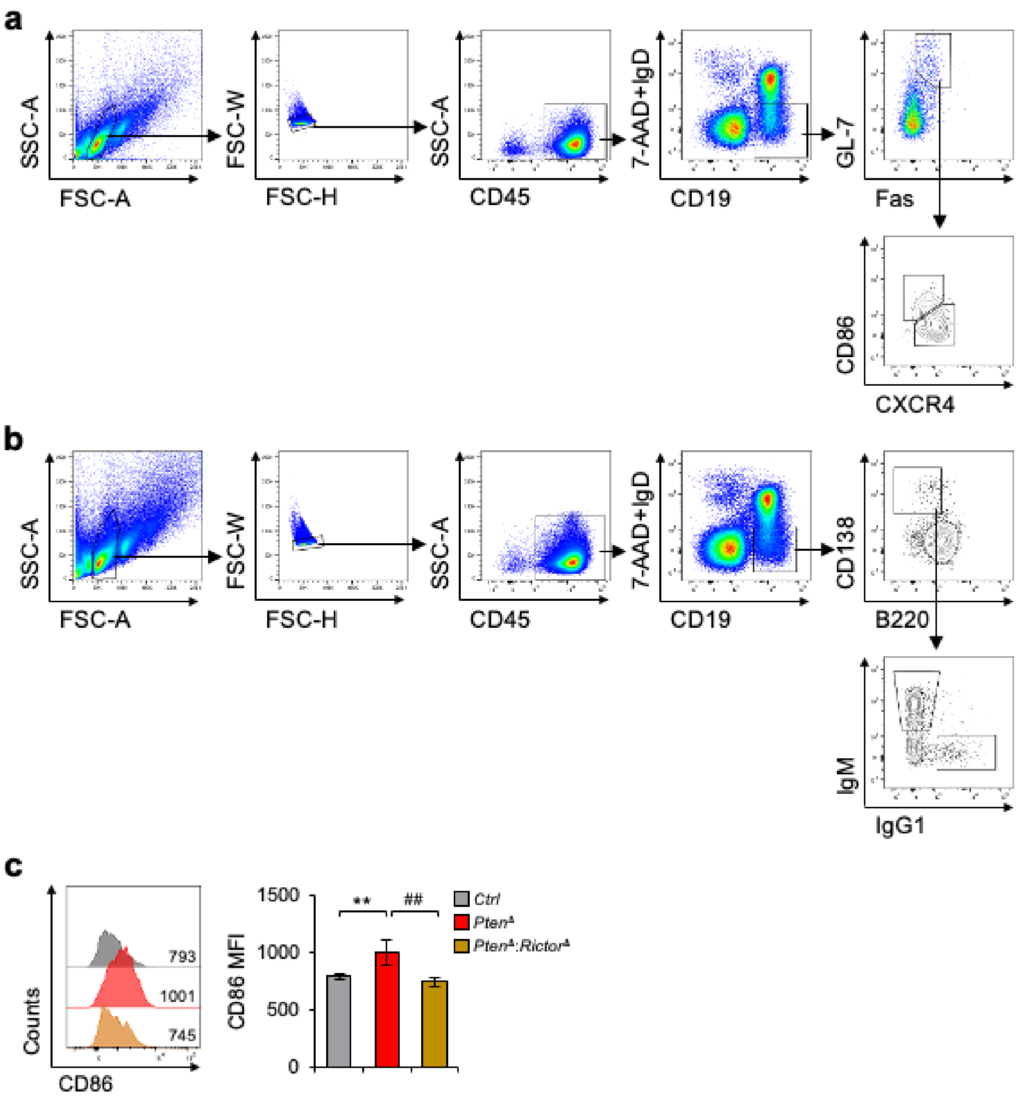

Thapa and Lee *et al.* Supplemental Figure S7

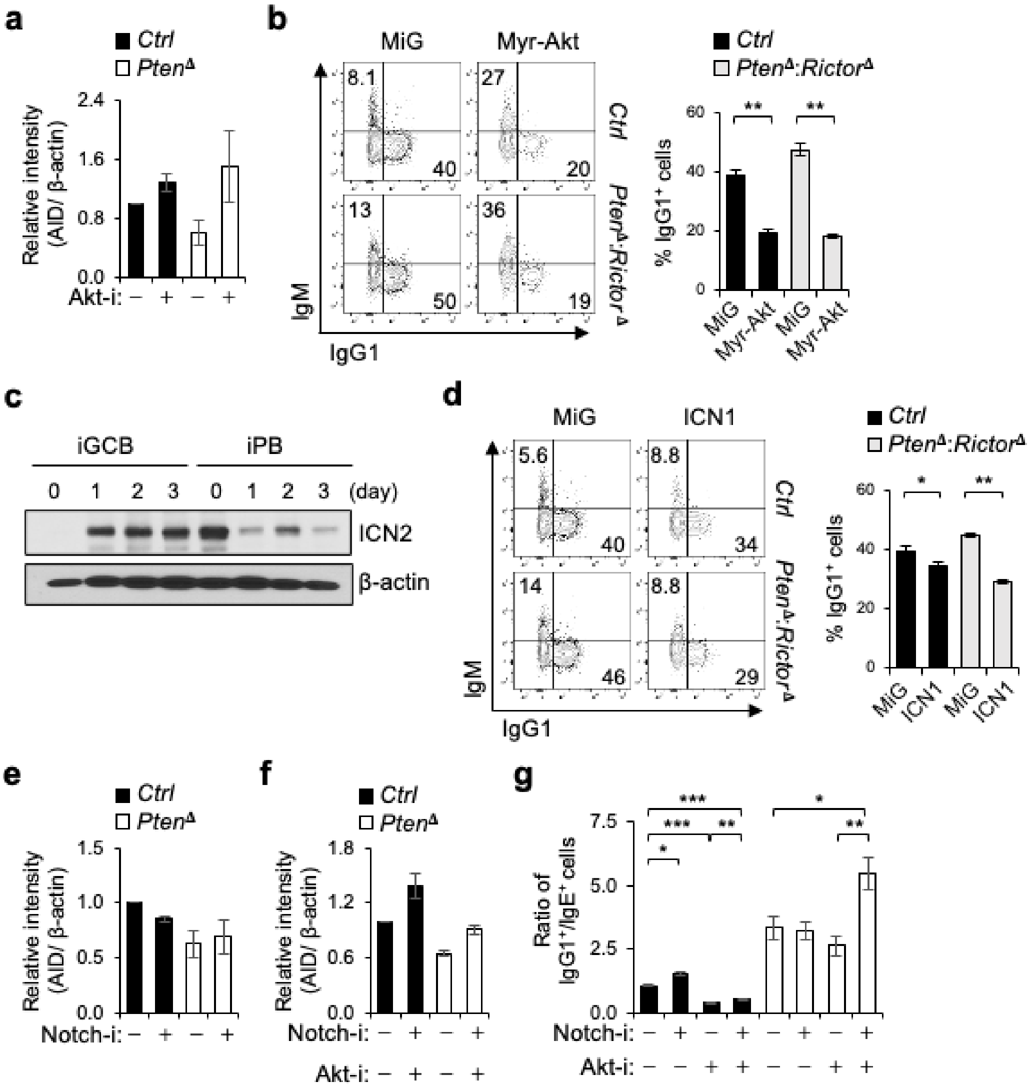

Supplement: Supplementary Table 1 — Primer sequences used in quantitative real-time PCR analysis. [file Presentation1.pdf]
